# Chamaejasmine B Induces the Anergy of Vascular Endothelial Cells to VEGFA Pro-angiogenic Signal by Autophagic Regulation of VEGFR2 in Breast Cancer

**DOI:** 10.3389/fphar.2017.00963

**Published:** 2018-01-22

**Authors:** Qi Li, Xiaoxi Kan, Jie Yin, Lidong Sun, Yajie Wang, Yujie Li, Qing Yang, Hongbin Xiao, Ying Chen, Xiaogang Weng, Weiyan Cai, Xiaoxin Zhu

**Affiliations:** ^1^Institute of Chinese Materia Medica, China Academy of Chinese Medical Sciences, Beijing, China; ^2^School of Traditional Chinese Medicine, Capital Medical University, Beijing, China; ^3^Key Laboratory of Separation Science for Analytical Chemistry, Dalian Institute of Chemical Physics, Chinese Academy of Sciences, Dalian, China

**Keywords:** Chamaejasmine B, anti-angiogenesis chemotherapy, breast cancer, autophagy, VEGFR2

## Abstract

The neovascularization functions essentially for malignant upgrading and predicts poor prognosis in multiple cancers, which make it the highly effective strategy for clinical treatment. Unfortunately, the known anti-angiogenic therapies show low effectiveness against breast cancer. Recently, rebalancing the pro-angiogenic property in microenvironment shows great advantages and attracts increasing attention for breast cancer treatment. Herein, we for the first time reported that Chamaejasmine B (ICJ), extracted from *Stellera chamaejasme* L., possessed potent anti-angiogenic effect in breast cancer. By Transwell, tube formation and aortic-ring assays, ICJ efficiently suppressed the neovascularization potential in tumor-HUVEC co-culture model. In Matrigel plug assay, the efficacy of ICJ was further identified *in vivo*. Mechanistically, with little influence on HUVEC apoptosis, ICJ obviously induced autophagy as proved by the elevated LC3I/II ratio, dotted distribution of LC3 and upregulated Beclin-1. Moreover, by associating with LC3 and in turn, inhibiting the level of VEGFR2, the anti-angiogenesis efficacy was closely dependent on the initiation of autophagy. Above results proved that, by attenuating the pro-angiogenic communication through VEGFR2, ICJ is a novel angiogenic inhibitor and will be a promising supplement for anti-angiogenic chemotherapy for breast cancer.

## Introduction

Breast cancer is the most common cancer among females worldwide and estimated to account for 29% of the total new cases and 14% of the total deaths in 2016 (Siegel et al., 2016). Notably, metastasis contributes to more than 90% of breast cancerous death (Nguyen et al., 2009; Talmadge and Fidler, 2010; Massagué and Obenauf, 2016). The importance of angiogenesis in metastasis has been firstly noticed in 1982 (Jensen et al., 1982). Clinically, angiogenesis in breast cancer is an early event and the microvessels density (MVD) is the key risk factor for metastasis of ductal carcinoma *in situ* and the predictor of poor prognosis (Weidner et al., 1991; Bielenberg and Zetter, 2015; Pei et al., 2017). More importantly, angiogenesis is far more than a metastasis helper; it also functions essentially in multiple malignant events such as oxygen supply (Strese et al., 2013), energy metabolism, inflammatory regulation (Fukumura et al., 2016) and chemotherapy resistance (Bottsford-Miller et al., 2012).

Physiologically, angiogenesis is regulated by a complicated and balanced network mainly consisting of VEGFs (Ellis and Hicklin, 2008; Domigan et al., 2015), PIGF, Angiopoietin-1/2, TSP-1/2 (Carmeliet and Jain, 2011). In contrast, in malignant condition, the angiogenic balance is perturbed and neovascularization is pathologically activated. Notably, not limiting to VEGFA, majority of pro-angiogenesis factors can interact with VEGFR2 (Duda, 2012) and by such mechanism, it functions as the molecular hub responsible for the integration of pro-angiogenic signals in tumor microenvironment.

Indicated by the essential roles of angiogenesis in cancer progression, as early as 1970's, Folkman had proposed that angiogenesis is a feasible target in anti-tumor therapy (Folkman, 1971; Sainson and Harris, 2007). Concentrating on the regulation of VEGFA secretion or VEGFR kinases activities, in the following 20 years, serials of anti-angiogenic drugs have been developed and showed great advantages in chemotherapy. Unfortunately, as represented by Bevacizumab, most of the current drugs showed limitation in breast cancer treatment (Robert et al., 2011; Bergh et al., 2012; Varinska et al., 2015). Therefore, identifying novel effective agents targeting any new angiogenic mechanism is extremely urgent and necessary. Recently, accumulating evidences showed that autophagy, a conserved catabolic degradation process, regulates tumor angiogenesis through the interaction between LC3 and angiogenic inducers, such as VEGFR2 and HIF-1α (Liu et al., 2012; Kumar et al., 2013). This findings provided another novel but reliable target for tumor angiogenic intervention.

Chamaejasmenin B (ICJ), a flavonoids from *Stellera chamaejasme* L, has been identified as a potent tumor toxic and anti-metastatic candidate in our previous study (Li et al., 2016). Literature reported that many chemotherapeutic drugs exerted anti-angiogenic activity in much lower concentrations than that of killing tumor cells (Pathania et al., 2015). Leading by this, the study was designed to prove that ICJ may negatively regulate angiogenesis in breast cancer. In tumor-vascular endothelial cell interaction model, our results pharmacologically identified a novel target for anti-angiogenic drug design and provided a promising compound which is effective for both metastasis and neo-vascularization in breast cancer.

## Materials and methods

### Cell culture and reagents

Lyophilized powder ICJ was extracted and purified by Dalian Institute of Chemical Physics, Chinese Academy of Sciences. The powder was dissolved with normal saline containing 10% DMSO and 1% Tween-80. The primary antibodies of LC3II, Beclin-1, VEGFR2 and β-actin were purchased from Sigma-Aldrich (USA), Abcam (Britain), Cell signaling Technology (USA) and Santa Cruz Biotechnology (USA) respectively. Matrigel™ was purchased from BD Biosciences (USA); the Annexin V-FITC/PI apoptosis and Caspase-3 detection Kits were purchased from CWBIO (China); Beclin-1 shRNAs were purchased from Genechem (China). The breast cancer cell line MDA-MB-231 and 4T1 were purchased from American Typical Collection Center (USA), Human umbilical vein endothelial cell (HUVEC) was donated by Dr. Liwei Gu from Institute of Chinese Materia Medica. Cells were cultured in RPMI 1640 containing 10% fetal bovine serum.

### Tumor cell-vascular endothelial cells co-culture and conditional cultural medium transferring models

**Figure 1:**
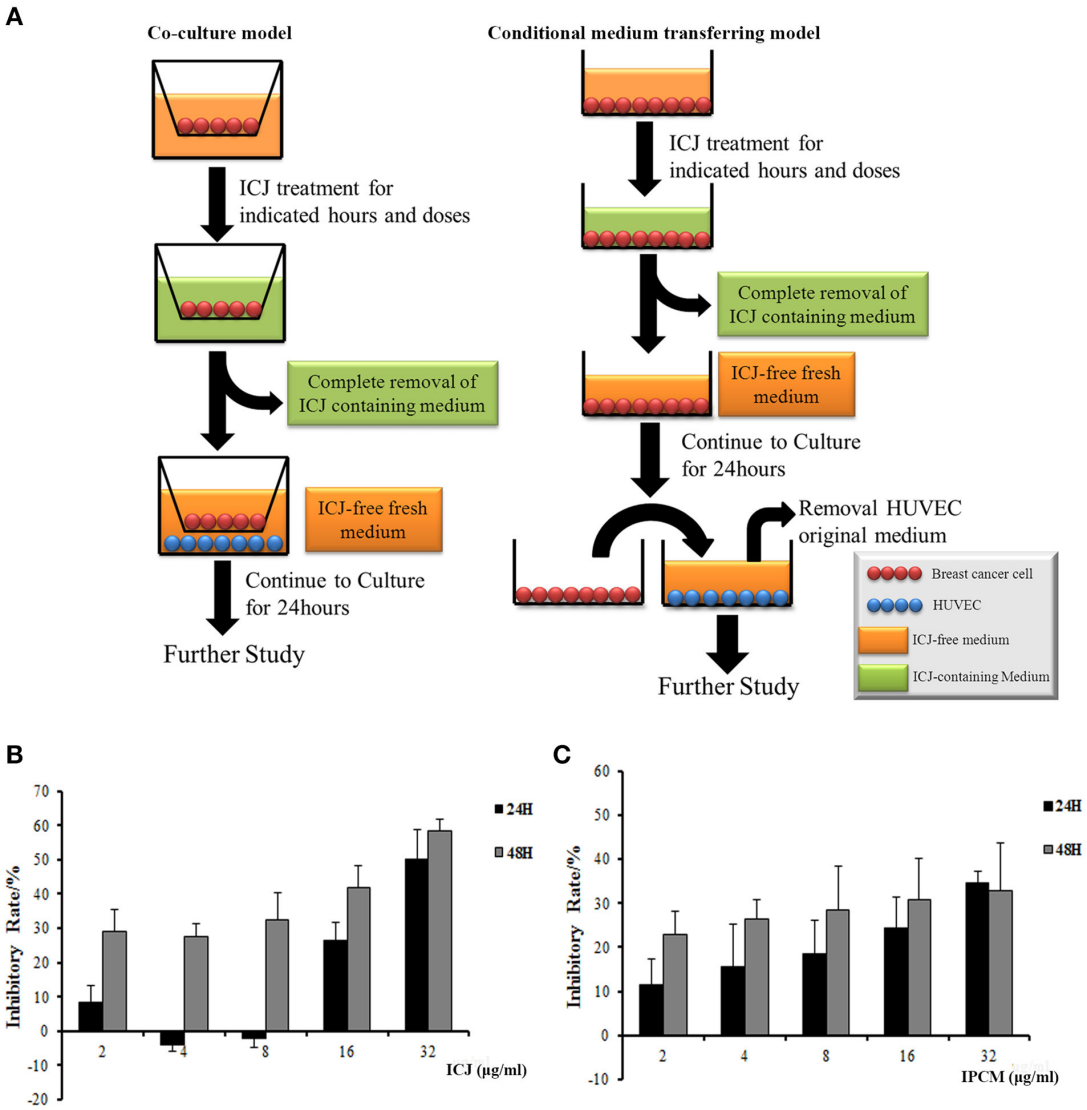
The establishment of experimental models and dose identification for *in vitro* study. **(A)** *In vitro* models for efficacy identification of ICJ. The left column shows the co-culture model and the right one is the conditional medium transferring model. **(B,C)** Detection of cell proliferation level in MDA-MB-231 **(B)** and HUVEC **(C)** incubated with ICJ and IPCM respectively.

In these two models shown in Figure 1A, instead of the direct treatment of ICJ on HUVEC, all the experiments were performed after the drug containing medium has been completely removed: (1) In co-culture system, MDA-MB-231 was seeded on the upper chambers (0.4 μm pore size) and treated with different concentrations of ICJ for 24 h (indicated as “IP-dose” in figures, which is the abbreviation for ICJ Pretreated-dose). Then, HUVEC was paved onto the lower cultural surface after the ICJ-containing medium has been totally replaced with the fresh one (ICJ-free medium). (2) The conditional medium transferring model was used as an alternative method in certain experiments such as MTT or aortic ring assay. The protocol was basically the same as the one in co-culture model except that the ICJ-free conditional medium was transferred from tumor cells to another cultural system. “IPCM-dose” (Abbreviated for ICJ Pretreated Conditional Medium) mentioned in the results means the conditional medium after ICJ treatment with indicated doses for 24 h.

### Endothelial cell capillary-like tube formation assay

Matrigel was liquefied at 4°C and pipetted into precooled 96-well plates (50 μl/well) and polymerized for 30 min at 37°C. HUVEC from ICJ-pretreated co-culture model was paved onto the surface of the Matrigel (2.5 × 10^4^ per well) and observed for 7 h. The tubular networks were quantified by the tube sprouting rates. Tube sprouting rate (%) = (sprouted cells/ total cells) × 100.

### Rat aortic ring assay

Eighty microlter/well diluted Matrigel (1:1 in opti-MEM) was precoated on 96-well plate and solidified for 30 min. Aortas isolated from 6-week-old male Sprague–Dawley rats were cleaned and cut into rings (circumference: 1~1.5 mm). The aortic rings were placed onto the wells and sealed with 50 μl diluted Matrigel. The ICJ-pretreated conditional medium was added to the wells in the presence of VEGF_165_ (Peprotech, USA). The conditional medium was changed every 2 days. After 8 days, the microvessels sprouted from the rings were stained with MTT and imaged (100× magnifications).

### Matrigel plug assay *in vivo*

0.5 ml of growth factor-free Matrigel in the presence or absence of 4T1 cells (1 × 10^5^) containing 200 ng VEGF165 and 20 U heparin was transplanted sub-cutaneously into the ventral area of 6-week-old C57/BL6 mice. The mice were daily intraperitoneally injected with 30, 150, and 750 μg/kg of ICJ. After 7 days, animals were sacrificed and the plugs were weighted and fixed for further detection. Randomly selected samples were prepared for CD146 immunohistochemical analysis and the hemoglobin content of plug was quantified through Drabkin's reagent (Sigma-Aldrich, USA).

### Orthotopic model of breast cancer

1 × 10^4^ 4T1 cells were injected into the fourth mammary fat-pad of 8-week-old female Balb/c mice. When tumors became touchable, the mice were grouped through average tumor size (10 mice/group). The mice were daily intraperitoneally injected with ICJ (30, 150, 750 μg/kg). At the 26th day, the primary tumors were surgical removed and fixed for CD146 IHC staining. The procedure of animal experiment was approved by Animal care and Welfare Committee of Institute of Chinese Materia Medica and the care of laboratory animal and the animal experimental operation was carried out in accordance to the Beijing Administration Rule of Laboratory Animal (shown in Table S2).

### RT-PCR assay

The mRNA was extracted by TRIzol reagent. The cDNA library was reverse-transcribed using the RevertAid First Strand cDNA Synthesis Kit (Fermentas, Canada). The protocol for the cDNA synthesis was: 45°C for 1 h followed by 70°C for 5 min. The primers for VEGFA detection were: Forward 5′-CACACAGGATGGCTTGAAG-3′; Reverse 5′-CTTGCCTTGCTGCTCTACC-3′. The GAPDH primers were: Forward 5′-CAAGGTCATCCATGACAACTTTG-3′; Reverse 5′-GTCCACCACCCTGTTGCTGTAG-3′.

### Transmission electron microscopic observation

HUVEC (1 × 10^6^) was fixed with 2.5% glutaraldehyde and postfixed with 2% osmium tetroxide. Followed by sequential steps of washing, dehydration, embedding and cut to ultrathin pieces, the samples were placed on uncoated copper grids and electron stained with lead citrate and uranyl acetate. The images (30,000×) were collected by 1400 transmission electron microscopy (JEOL Ltd., Japan).

### Co-immunoprecipitation analysis

Cells were lysed with proper volume of lysis buffer containing protease-inhibitor cocktail (100×, Roche, Swiss). The cell lysates were incubated with LC3 antibody under agitation at 4°C overnight. Protein G Sepharose 4 fastflow (GE healthcare, USA) was added into the lysate to bind with antibody for 4H. The bead was separated from the mixture by centrifuged at 12,000 g. Wash the bead 3~4 times using lysis buffer and then 15 μl of 2×SDS loading buffer was added for western blot analysis.

### Beclin-1 silencing

Silencing sequence specifically targeting Beclin-1 and scramble control sequence (shown in Table S1) were inserted into GV248 vectors respectively. Then, the constructed plasmids were amplified in *E. coli*. and prepared by Endo-free plasmid maxi-extraction Kit (Tiangen Boitech, China). After purification and sequencing identification, the plasmids were transfected into HUVEC by Lipofectamine® 3,000 according to the manufacture's instruction (Invitrogen, USA).

### Statistical analysis

All the results shown in the figures were performed at least 3 independent repeats. All the quantitative data shown in this manuscript was presented as arithmetic means ± standard deviation. Statistical analysis was calculated with SPSS 17.0 software and one-way ANOVA was performed. *P* < 0.05 represented the statistical significance (^*^p < 0.05, ^**^p < 0.01, ^***^p < 0.001).

## Results

### Experimental model establishment and dose identification *in vitro*

In order to specifically clarify whether ICJ regulates the interaction between tumor cells and vascular endothelial cells, we established the tumor-HUVEC co-culture and conditional medium transferring models. The protocol was detailed in “Material and Methods” section (Figure 1A). Notably, in those systems, the direct influence of ICJ on HUVEC alone has been successfully excluded. Therefore, the interaction changes between ICJ-pretreated tumor cells and HUVEC cells can be explicitly identified.

Next, MTT assay was performed to determine the safety dose and to exclude the toxic effect of ICJ or ICJ pretreated conditional medium (IPCM). Results showed that the inhibition rates increased in a dose- and time-dependent manner. Less than 8 μg/ml of ICJ and ICJ-pretreated conditional medium exerted little influence on cell survival and proliferation both in MDA-MB-231 and HUVEC (Inhibitory rate<20%), therefore, this condition was chosen for further study (Figures 1B,C).

### ICJ attenuates pro-angiogenic effect of breast cancer cells *in vitro*

**Figure 2:**
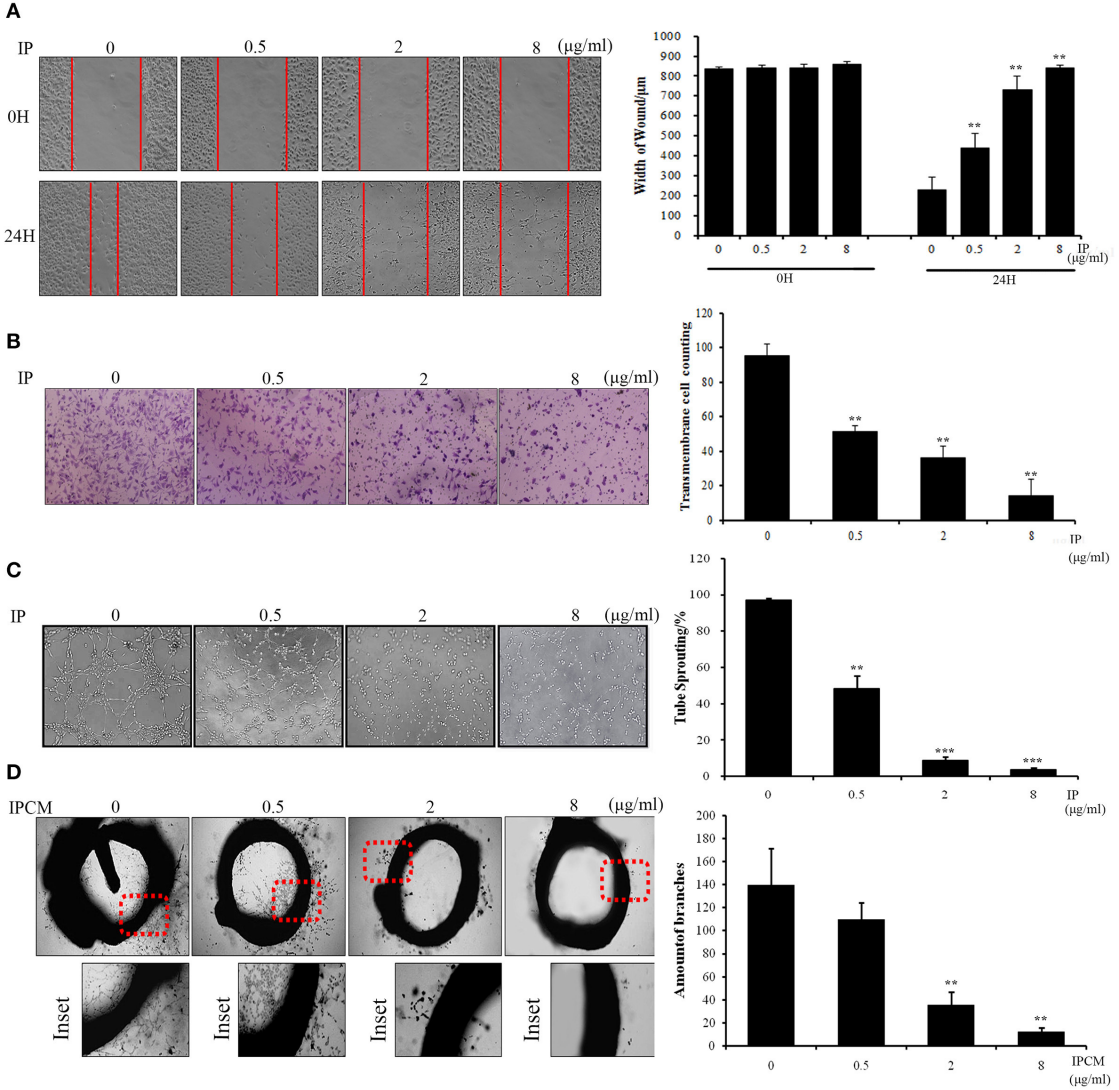
The anti-angiogenesis efficacy of ICJ. **(A)** Wound healing assay in ICJ-pretreated co-culture model. Images were collected on 0 and 24 h. The wound width was measured in 3 randomly selected fields (100×). **(B)** Transwell migration assay in HUVEC. The pictures were taken at 18H and transmembraned cells were quantified in 5 randomly selected fields (200×). **(C)** Capillary-like tube formation assayin ICJ-pretreated HUVEC-tumor co-culture model. The result was quantified by tube sprouting rates calculated in 5 randomly chosen fields (200×). **(D)** The rat aortic ring analysis. The microvessels were count to evaluate the angiogenesis level in tumor microenvironment. ^*^p < 0.05, ^**^p < 0.01, ^***^p < 0.001.

To phenotypically assess the anti-angiogenic activity of ICJ in the tumor-endothelial cell interaction model, we firstly performed wound healing and Transwell assays (Figures 2A,B) to evaluate HUVEC migration potential, which is essential for neo-vascularization. The results clearly exhibited that the migration inhibitory effect can be dose-dependently detected in co-culture model. As shown in the figure, in IP-8 ug/ml group, the amount of transmembrane cells was significantly decreased from (95.6 ± 6.58) to (25.0 ± 5.24) and the wound closure width was increased from (231.11 ± 60.35) to (842.56 ± 12.65) μm.

Accordingly, the anti-angiogenic capability of HUVEC was further confirmed by capillary-like tube formation assay (Figure 2C). The typical tubular-like structures can be observed in control group, whereas the capillary-like structure was significantly and dose-dependently destroyed in ICJ pretreatment group. Additionally, the rat aortic ring assay, which recapitulated the key steps of angiogenesis, also demonstrated that the length and density of new microvessels sprouting from the aortic rings were markedly decreased in ICJ-pretreated conditional medium (Figure 2D). Collectively, the results suggested the anti-angiogenic potential of ICJ in tumor-HUVEC co-culture model *in vitro*.

### ICJ suppresses breast cancer angiogenesis *in vivo*

**Figure 3:**
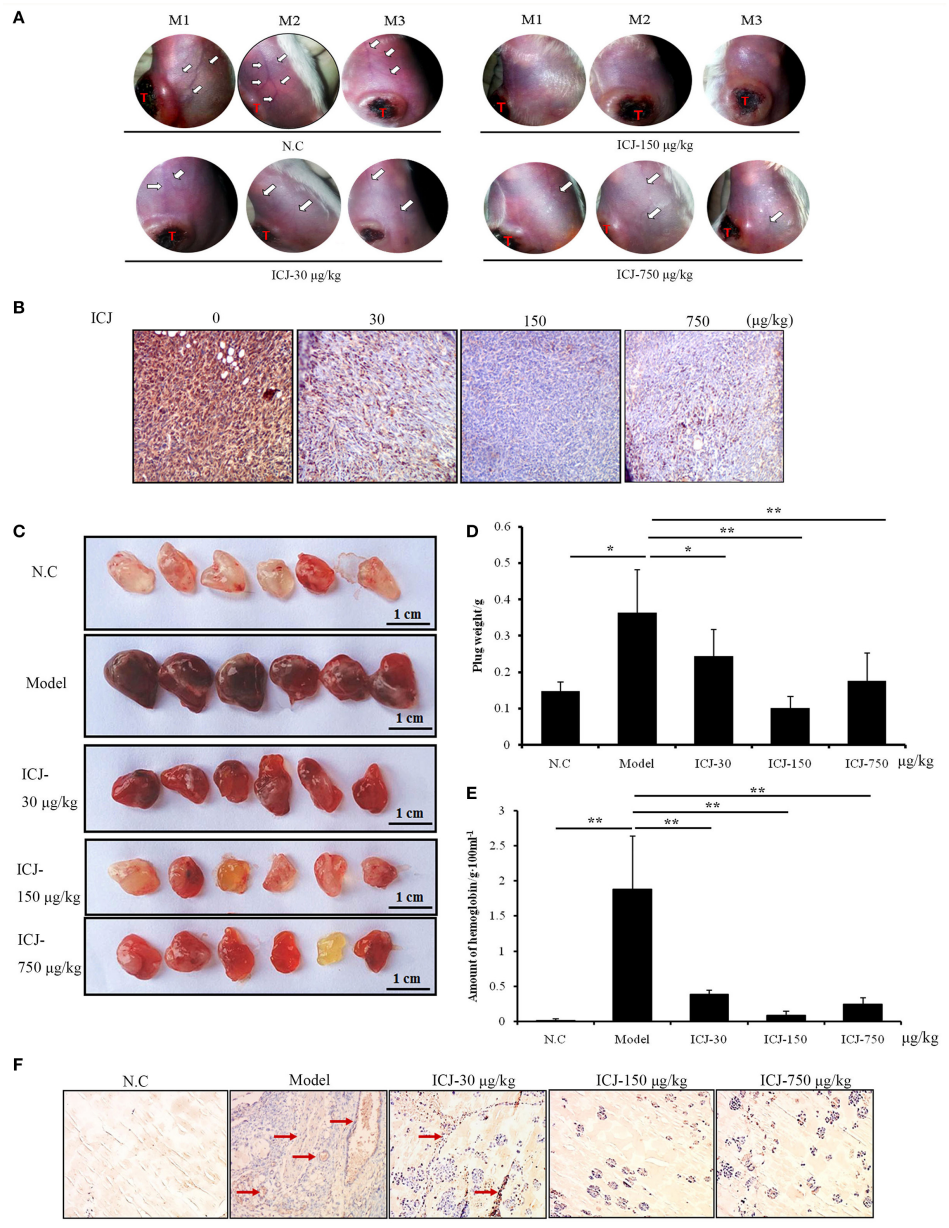
The anti-angiogenesis efficacy of ICJ *in vivo*. **(A)** The observation of tumor-surrounding abdominal vessels in orthotopic model. The pictures were taken from 3 mice in each group at 26th day after modeling. The red letter “T” indicates the primary tumor; the white arrows were pointed to the abdominal vessels. **(B)** IHC analysis of primary tumors *in vivo*. Tumor tissues were obtained from the orthotopic model (three mice per group) and CD146 was detected to verify the neovascularization level. **(C)**Matrigel plug assay. The plugs samples were analyzed on the 7th day after modeling. **(D)** The weight of plugs in each group (*n* = 6). **(E)** The hemoglobin content of plugs. 3 randomly selected plugs from each group were homogenized for this detection. **(F)** IHC analysis of Matrigel plugs. The plugs were fixed and the expression level of CD146 was detected by IHC. Red arrows indicated the neovascularized section. (100× magnification). ^*^p < 0.05, ^**^p < 0.01, ^***^p < 0.001.

Firstly, to further identify the anti-angiogenic activity of ICJ *in vivo*, orthotopic breast cancer model was used. In this model, 4T1 breast cancer cells were orthotopically transplanted into the 4th breast fat pads in female Balb/c mice. After tumor growing for 26 days, the morphological observation of the tumor-surrounding tissues clearly suggested that ICJ obviously inhibited the neo-vascularization sprouting from the primary tumors. The density and the area of new vessels network were both decreased comparing to the negative control mice (Figure 3A). Additionally, to histologically verify our study, CD146, a diagnostic indicator of breast cancer and the specific biomarker for malignant angiogenesis (Zeng et al., 2012, 2014), was stained in primary tumor tissues. Result showed that CD146 was extensively expressed in non-ICJ-treated group, whereas the positive signal of CD146 was remarkably decreased in drug administrated mice, especially in 150 μg/kg group (Figure 3B). The above results collectively proved that ICJ is sufficient to reverse the elevated angiogenesis in primary tumor tissues in orthotopic animal model.

Leading by this, Matrigel plug assay was also performed. Comparing with orthotopic model, Matrigel plug assay specifically and directly reflects the neo-vascularization potential *in vivo*. In our study, tumor-containing plugs were transplanted in C57/BL6 mice and exposed to different doses of ICJ. The results clearly exhibited that, in model group, 4T1 cells successfully induced angiogenesis in Matrigel plugs with the increased plug weight, the crimson appearance and the high content of hemoglobin. In contrast, the ICJ exposed plugs showed an obvious reduction of vascularization. The plug weight was decreased and the color changed to pale red with less hemoglobin concentration (Figures 3C–E).

Consistently, The IHC images from Matrigel plugs further proved the decreased expression of CD146 in the presence of ICJ (Figure 3F). In model group, high level of CD146 expression and MVD can be clearly observed. In the opposite, in addition to the lower MVD, the expression level of CD146 is dramatically decreased in drug-exposed groups. Moreover, the invasive growth pattern of tumor cells in non-treated group was greatly changed by ICJ, which featured the round and isolated clones. Collectively, through the above-mentioned results, we have successfully revealed the anti-angiogenic effect of ICJ *in vivo*.

### ICJ selectively induced autophagy of HUVEC in tumor microenvironment

**Figure 4:**
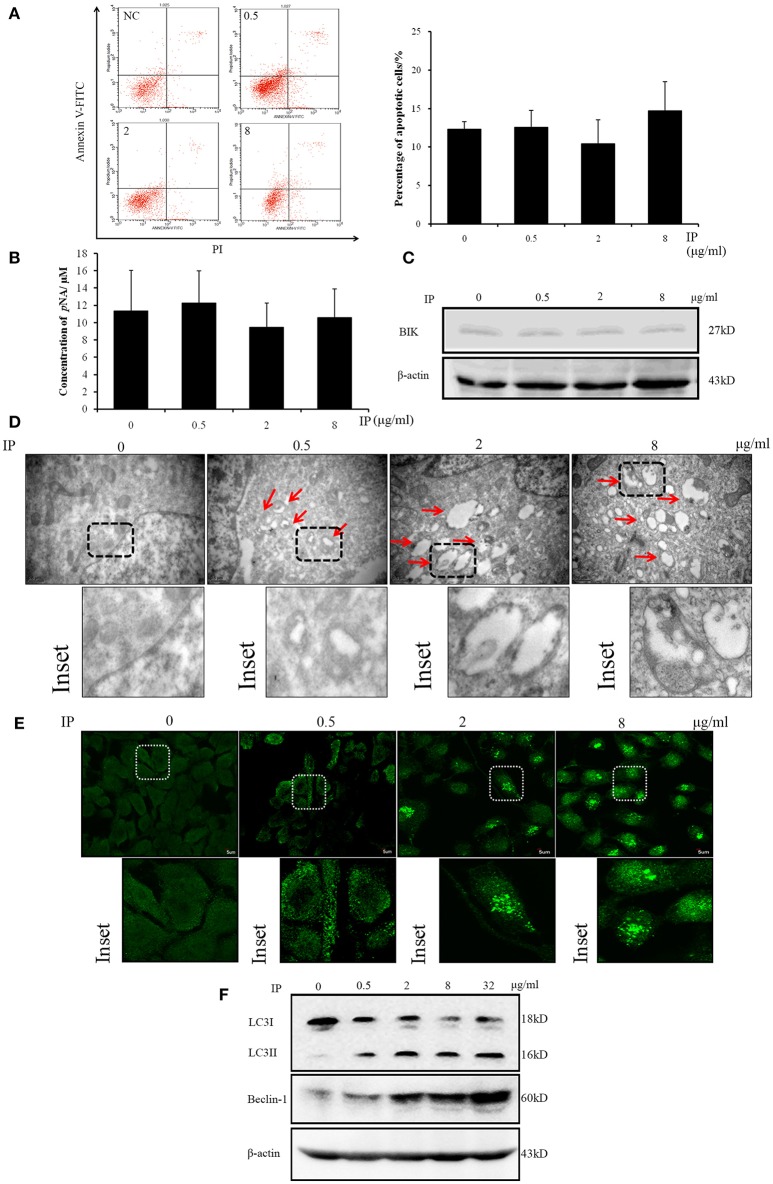
Apoptosis and autophagy analysis of HUVEC in ICJ-pretreated co-culture model. **(A)** Annexin V-FITC/PI staining and flow cytometry analysis of HUVEC. The apoptotic levels were quantified at 24 h in ICJ-pretreated co-culture model. **(B)** Caspase 3 activity detection in HUVEC. As shown in the column chart, the activities of Caspase 3 were approximately unchanged among groups. **(C)** Western Blot analysis of BIK. The similar expression intensity can also be detected in HUVEC from co-culture model. **(D)** Transmission electron microscopic observation of HUVEC. The autophagosomes (indicated by the red arrows) can be clearly observed in the picture (30 k× magnification). **(E)** Confocal microscopic observation of LC3 dotted localization of HUVEC (600× magnification). **(F)** Detection of the autophagic biomarkers (LC3I/II and Beclin-1) in HUVEC by Western Blot analysis.

In the mechanism study, we firstly analyzed whether ICJ could induce apoptosis of HUVEC in co-culture model. Through Annexin V-FITC/PI staining, it was found that apoptosis-rate kept unchanged among groups (Figure 4A). In addition, the key apoptotic regulators were also detected. As shown in the figure, the activity of Caspase 3 and the expression of BIK maintained at the comparable levels between negative control and ICJ pretreated groups (Figures 4B,C). Taken together, these results indicated that the angiostatic activity of ICJ was independent of apoptosis.

Interestingly, in the next study, we used transmission electron microscopy to observe the ultra-structural alterations of HUVEC in co-culture model pretreated with ICJ. Surprisingly, large amounts of autophagosomes appeared in response to ICJ (Figure 4D). To further validate this efficacy, immunofluorescence assay was used to detect the sub-cellular localization of LC3 in HUVEC. Different from the diffused cytoplasmic distribution in untreated cells, LC3 changed to punctate localization by ICJ pretreatment for 24 h (Figure 4E), which provided another evidence for the autophagy-inducing effect of ICJ. Moreover, the same conclusion has been supported by western blot analysis (Figure 4F and Figures S1, S3, S4) showed that the expression of LC3II and Beclin-1, two markers of autophagy, were both upregulated in HUVEC collected from ICJ pretreated co-culture model.

### The blockage of autophagy rescues the ICJ-impaired angiogenesis

**Figure 5:**
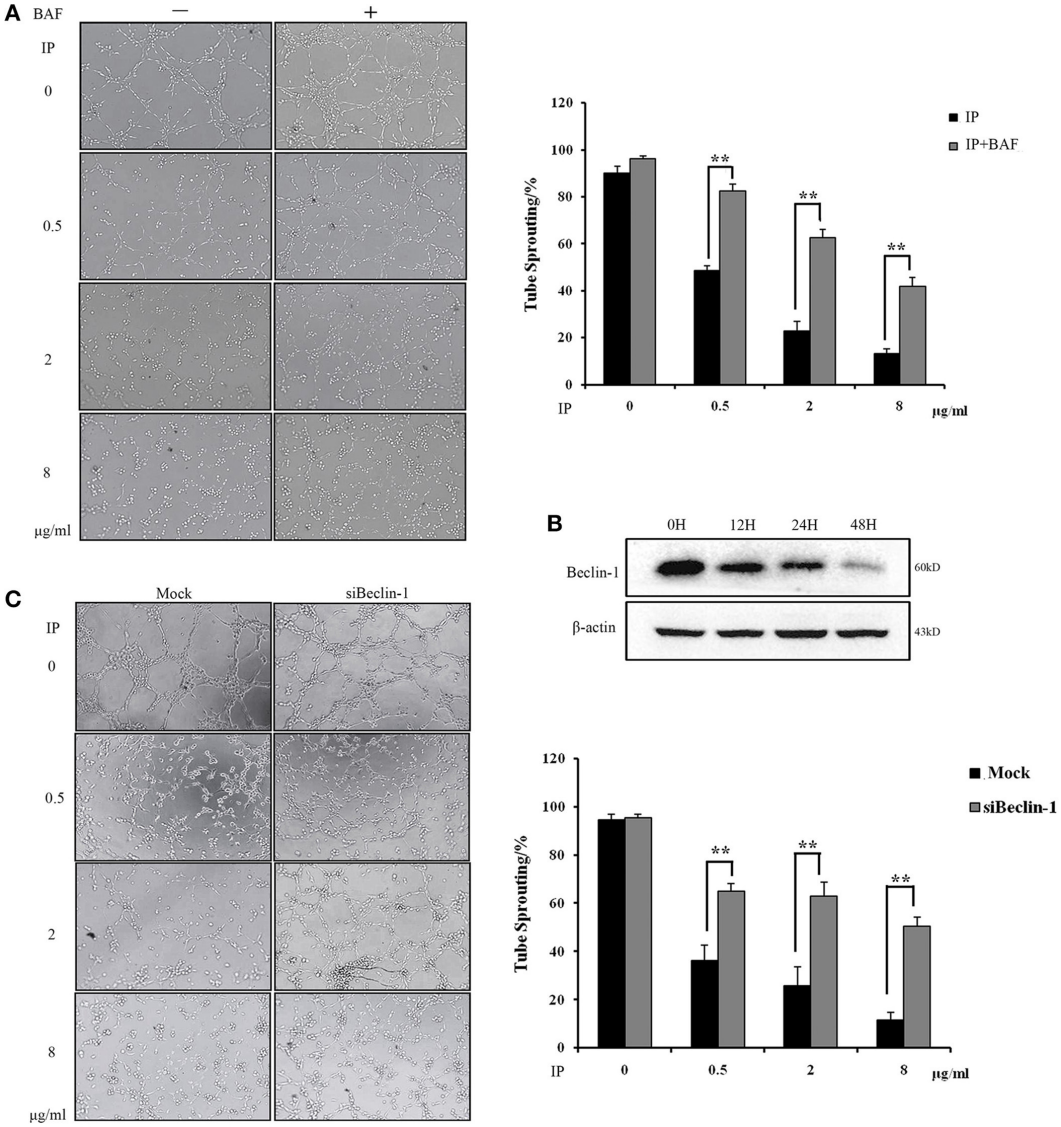
Autophagy induced by ICJ attenuated angiogenesis *in vitro*. **(A)** The capillary-like tube formation assays in HUVEC in the presence or absence of BAF. In BAF^+^ group, 100 nM BAF was incubated with HUVEC for 24 h. **(B)** Silencing efficiency test of Beclin-1. Western Blot showed that Beclin-1 expression was time-dependently silenced**. (C)** The capillary-like tube formation assays in Beclin-1 deficient HUVEC (100×). Cells were all collected from ICJ-pretreated co-culture model. Results indicated that deficiency of autophagy reversed the efficacy of ICJ. ^*^p < 0.05, ^**^p < 0.01, ^***^p < 0.001.

Based on the dual effects of ICJ on the regulation of autophagy and angiogenesis in HUVEC, we next tried to examine the dependency between these two procedures. Firstly, Bafilomycin A1 (BAF), the inhibitor of autophagy (Yuan et al., 2015), was used and the tube formation assay was performed under the autophagy deficient condition. As presented in Figure 5A, the fractured network induced by ICJ pretreatment was obviously recovered by BAF. Additionally, it is well proved that Beclin-1 plays pivotal role in the initiation of autophagy (Kang et al., 2011). Take this into account; Beclin-1 was silenced in HUVEC (Figure 5B and Figures S2, S5, S6) and the enhanced tube formation potential was also observed (Figure 5C). Compared with Mock groups, the tube sprouting rates were 2~3 times higher in Beclin-1 silenced groups, which further proved that ICJ-mediated autophagy was responsible for the inhibition of breast cancer angiogenesis.

### ICJ inhibits malignant angiogenesis by autophagic interaction between LC3 and VEGFR2

**Figure 6:**
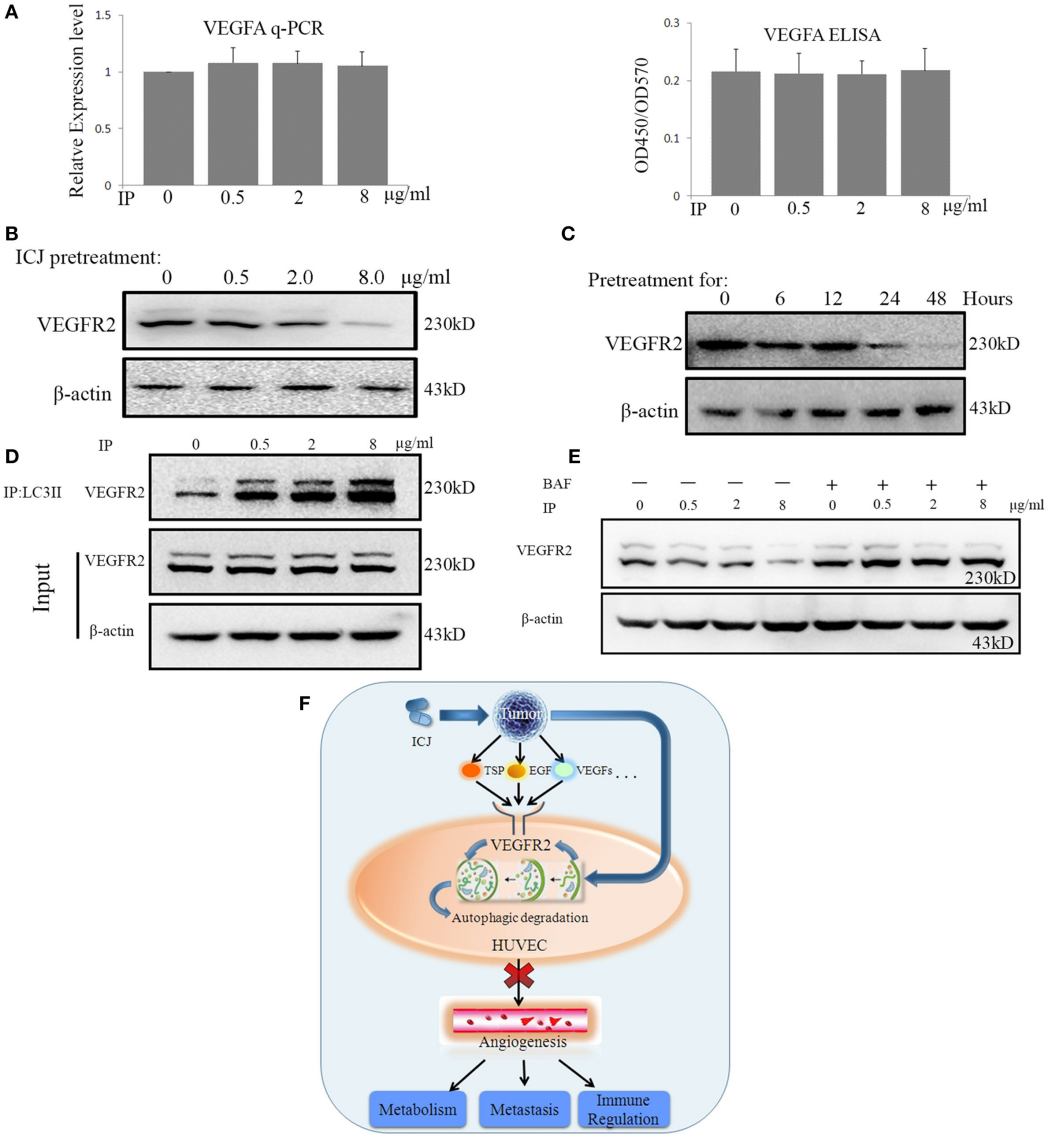
ICJ induced the Autophagic reduction of VEGFR2 in HUVEC. **(A)** VEGFA detection by Realtime-PCR and ELISA in co-culture model. Results showed that VEGFA maintained at the comparable level among all the groups. **(B)** Western analysis of VEGFR2 in tumor-HUVEC co-culture model pretreated of ICJ for 24 h. **(C)** The time-course detection of VEGFR2 by 8 ug/ml of ICJ pretreatment. It can be seen that the VEGFR2 expression was time-dependently decreased and transient treatment of ICJ could not induce the downregulation of VEGFR2. **(D)** Co-immunoprecipitation analysis in HUVEC from co-culture model. Results showed that ICJ promoted the interaction between LC3II and VEGFR2. IP, Immunoprecipitation; IB, Immunoblotting. **(E)** Western blot analysisofVEGFR2 in BAF treated or non-treated HUVEC. **(F)** The graphic summary for the anti-angiogenic study of ICJ. As the master executor for angiogenesis, vascular endothelial cells sense and integrate he pro-angiogenesis signals from tumor cells and phenotypically enhance the sustained neovascularization. During such communication, VEGFR2 takes the central position and becomes the key participant bridging tumor and endothelial cells. Importantly, as proved by our study, VEGFR2 is right the molecular target for the anti-angiogenesis effects of ICJ. By some unknown mechanism, ICJ activates the autophagic promotion signals from tumor cells and enhances the autophagic degradation of VEGFR2 in HUVEC, which result in the blockage of the proangiogenesis crosstalk between tumor and HUVEC.

Based on the above result, we wondered how ICJ-mediated autophagy negatively controlled angiogenesis. We initially focused on the detection of VEGFA expression. Unexpectedly, Real-time PCR and ELISA analysis showed that ICJ barely influenced the VEGFA expression and secretion in HUVEC (Figure 6A), which indicated the efficacy of ICJ has little correlation with VEGFA.

As described in the introduction, VEGFR2 functions as the molecular hub for pro-angiogenesis signals. Inspired by this, we detected the VEGFR2 expression in HUVEC in co-culture system. The result showed that VEGFR2 was dose- and time-dependently influenced in co-culture system. Longer than 24 h of ICJ pretreatment was sufficient to inhibit the expression of VEGFR2 (Figures 6B,C and Figures S7–S9). This result for the first time revealed that ICJ, an angiogenic inhibitor, could regulate VEGFR2 expression in vascular endothelial cells.

Leading by such exciting evidence, we next make effort to further find the molecular explanations about the relationship between ICJ and VEGFR2 in co-culture models. As proved by other researches, the LC3-VEGFR2 interaction was negatively associated with the neovascularization. Moreover, the autophagic degradation of VEGFR2 is sufficient to induce the anti-angiogenic effect. We therefore postulated that the downregulated VEGFR2 was closely correlated with ICJ induced autophagy. Based on this, the physical interaction between LC3 and VEGFR2 was crucial for our study. In this assay, to avoid the interference from the expression change of VEGFR2 induced by long-term ICJ pretreatment, ICJ transient pre-treated co-cultured model was designed. By this model, the expression of VEGFR2 kept unchanged as identified by western blot analysis (ICJ pretreated to tumor cells for 6 h) (Figures 6C,D). Under such condition, co-immunoprecipitation assay showed that ICJ strongly increased the association between these two factors in comparison with untreated cell (Figure 6D and Figures S10, S11). This result clearly suggested that VEGFR2 was the potential target for ICJ induced autophagic degradation.

Additionally, to further verify the decrease of VEGFR2 induced by ICJ was dependent on autophagy, the autophagic inhibitor was used and the VEGFR2 expression was measured under autophagy deficient condition. As expected, the impaired expression of VEGFR2 was obviously reversed by BAF (Figure 6E and Figures S12, S13), which put another evidence to support the close relationship between autophagy and VEGFR2 in the presence of ICJ.

Taken together, our study molecularly proved that ICJ blocked the communication between tumor cells and HUVEC by inducing autophagic degradation of VEGFR2 and functionally inhibited angiogenesis in breast cancer microenvironment (Figure 6F).

## Discussion

As one of the malignant hallmarks, angiogenesis is proportional to tumor growth, proliferation, migration and metastasis (Hanahan and Weinberg, 2011; Vasudev and Reynolds, 2014). Studies further revealed that angiogenesis occurs surprisingly early in tumor progression. Even in non-invasive lesions, such as dysplasia and *in situ* carcinomas, angiogenesis can also be detected (Hanahan and Folkman, 1996; El-Kenawi and El-Remessy, 2013). Therefore, with wide treatment time window and little side-effect, drugs targeting devascularization are considered as one of the promising choice in tumor comprehensive treatment (Folkman, 2007; Sainson and Harris, 2007).

Currently, anti-angiogenic chemotherapies mainly focus on blocking VEGF:VEGFR signal pathways (such as Bevacizumab/Avastin®; Alvarez et al., 2011, Sunitinib Welti et al., 2012, and Sorafenib Devapatla et al., 2015) and enhancing the activity of endogenous angiogenesis inhibitors (such as angiostatin and endostatin) (Hutzen et al., 2014). Noteworthy, unlike other cancers, breast cancer is widely recognized as the tolerant type to the existed anti-angiogenesis therapies. Most angiostatic drugs targeting tyrosine kinase, such as Sorafenib, have failed to apply successfully (Robert et al., 2011; Bergh et al., 2012; Crown et al., 2013; Sun et al., 2014). Bevacizumab, the only approved anti-angiogenesis drugs in breast cancer, is not effective in all patients and has the potential risk of drug-resistance and severe side-effects (Varinska et al., 2015). Therefore, in our study, by identifying anti-angiogenic effects of ICJ in breast cancer, it will be beneficial to break the bottleneck of breast cancer treatment in the future.

The importance of microenvironment, as represented by the “Seeds and Soil” theory proposed in 1889 (Fidler, 2003), has drawn continuous attention over century. It has been widely accepted that the microenvironment was the decisive factor for tumor progression. Notably, a compelling body of evidence indicated that tumor cells, the “seeds,” did not only passively adapt to the soil, but took the initiatives to re-construct the microenvironment and made it more suitable for prosperous survival. Benefiting from the successful application of PD-1 and CTLA-4 monoclonal antibodies (Callahan and Wolchok, 2013), such field has been the hotspot for drug development. Angiogenesis is the most representative event in tumor microenvironmental reconstruction. The pro-angiogenesis property is formed on the basis of the communication between tumor and non-malignant cells from tumor microenvironments. Noteworthy, different from the most anti-angiogenesis research which directly observed the efficacy on vascular endothelial cells, our study focused on the mutual communication between tumor and vascular endothelial cells. By targeting tumor-specific signals, we revealed the blocking effects of ICJ in the pro-angiogenic signal transduction between tumor and vascular endothelial cells. These results reflected the selectivity of ICJ on the inhibition of malignant angiogenesis rather than the one under physiological condition (Figure 6F).

From the standpoint of drug research and development, there exists the undeniable fact for current anti-angiogenic research: their efficacy and intensity are far lower than expected clinically. Some *in vitro* qualified candidates show little effects *in vivo*. This largely limited the significance of anti-angiogenic research in certain types of cancers, especially in breast cancer. Under such circumstance, the efficacy evaluation *in vivo* are widely recognized as the indispensible part for anti-angiogenic drug research and development. In our study, we pay more attention to the *in vivo* evaluation of ICJ for their anti-angiogenic activity. In orthotopic and Matrigel plug assays, results consistently identified ICJ as an *in vivo* inhibitor for tumor-associated neo-vascularization, which ensure the future application of ICJ clinically.

Autophagy is well-known as the Janus in malignant cells. Especially the relationship between angiogenesis and autophagy have not reached a definitely conclusion. Our study identified the angiostatic association of autophagy induced by ICJ, which proved that regulation of autophagy could be a promising strategy in clinical breast cancer treatment.

More interestingly, as designed in our experiments, there weren't any physical contact between MDA-MB-231 and HUVEC, which means there must be one or some secretory protein(s) that bridged the communication between two cells and triggered autophagy in HUVEC. Identification of such soluble mediators is needed and will be essential for depicting the detailed molecular behaviors of ICJ in the crosstalk between autophagy and angiogenesis.

## Conclusion

By tumor-vascular endothelial cell interaction model, we for the first time reported that low-dose ICJ, with little cytotoxic effects, induces the anergy of HUVEC to VEGFA pro-angiogenic signals from tumor and downregulates VEGFR2 in an autophagy dependent manner. By such mechanism, ICJ specifically inhibited breast cancer angiogenesis. In addition, in our study, some indications have prompted that autophagic degradation of VEGFR2 is a feasible target for anti-angiogenic treatment in breast cancer.

## Author contributions

QL: contributed to the experiment design, experiment work, manuscript draft, and data analysis; XK: contributed to the experiment implementation and manuscript draft; JY and LS: assisted with manuscript modification and experiments *in vitro*; HX: prepared the experimental compound; YW, QY, and YL: participated in experiments *in vivo*; YC, XW, and WC: assisted with Co-immunoprecipitation analyses; XZ: took charge of the research design and coordinated the whole project; All authors reviewed the results and approved the final version of the manuscript.

### Conflict of interest statement

The authors declare that the research was conducted in the absence of any commercial or financial relationships that could be construed as a potential conflict of interest.

## Supplementary material

Table S1 The scramble control sequence and the silencing sequence targeting on Beclin-1.

Table S2 Application form for Welfare and Ethical Review in Animal Experimentation.

Figure S1 β-actin in Western blot of ICJ selectively induced autophagy of HUVEC.

Figure S2 β-actin in Western blot of the blockage of autophagy rescues the ICJ-impaired angiogenesis.

Figure S3 Detection of the autophagic biomarker, LC3I/II in HUVEC by Western blot analysis.

Figure S4 Beclin-1 in Western blot of ICJ selectively induced autophagy of HUVEC.

Figure S5 β-actin in Western blot analysis of VEGFR2 in tumor-HUVEC co-culture model pretreated of ICJ for 24 h.

Figure S6 Silencing efficiency test of Beclin-1. Western blot showed that Beclin-1 expression was time-dependently silenced.

Figure S7 β-actin in Western blot analysis of time-course detection by 8 mg/ml of ICJ pretreatment.

Figure S8 VEGFR2 in Western blot analysis of VEGFR2 in tumor-HUVEC co-culture model pretreated of ICJ for 24 h.

Figure S9 VEGFR2 in Western blot analysis of time-course detection of VEGFR2 by 8 mg/ml of ICJ pretreatment.

Figure S10 β-actin in Western blot analysis of co-immunoprecipitation analysis in HUVEC from co-culture model.

Figure S11 VEGFR2 in Western blot analysis of co-immunoprecipitation analysis in HUVEC from co-culture model.

Figure S12 β-actin in Western blot analysis of BAF treated or non-treated HUVEC.

Figure S13 VEGFR2 in Western blot analysis of BAF treated or non-treated HUVEC.

## Abbreviations

HUVEC: Human umbilical vein endothelial cell
ICJ: Chamaejasmine B
PIT: Post-ICJ-treatment
PICM: Post-ICJ-treated conditional medium.
