# Supplementary material for: Chamaejasmine B Induces the Anergy of Vascular Endothelial Cells to VEGFA Pro-angiogenic Signal by Autophagic Regulation of VEGFR2 in Breast Cancer
Source: Front Pharmacol. 2018 Jan 22;8:963. doi: 10.3389/fphar.2017.00963 (PMC5786572; doi:10.3389/fphar.2017.00963)
Supplement: Table S1 — The scramble control sequence and the silencing sequence targeting on Beclin-1. [file Table1.DOCX]

**Supplementary Table S1:** **The** **scramble control sequence and the silencing sequence targeting on Beclin1**

| **Name** | **Sequences** |
| --- | --- |
| Beclin-1 RNAi | GGAGCCATTTATTGAAACT |
| Scramble control | TTCTCCGAACGTGTCACGT |
