# Supplementary material for: Chamaejasmine B Induces the Anergy of Vascular Endothelial Cells to VEGFA Pro-angiogenic Signal by Autophagic Regulation of VEGFR2 in Breast Cancer
Source: Front Pharmacol. 2018 Jan 22;8:963. doi: 10.3389/fphar.2017.00963 (PMC5786572; doi:10.3389/fphar.2017.00963)
Supplement: Table S2 — Application form for Welfare and Ethical Review in Animal Experimentation. [file Table2.PDF]

编号：2015203

中国中医科学院中药研究所

动物实验福利伦理审查表（AN-T001）

申请单位或部门：中国中医科学院中药研究所					申请日期	2015.6.24
申请人相关信息	姓名	李琦	学历	博士	职称	助理研究员
	岗位证书编号		41907		联系电话	13260181996
	电子邮箱	qli@icmm.c.cn				

实验名称：狼毒宁B（ICJ）通过抑制血管生成抗肿瘤转移的体内药效药理实验

课题来源：☑ 国家课题，☐ 院级课题，☐ 所级课题，☐ 横向课题，☐ 其它

课题名称：以瑞香狼毒为代表的中药抗转移活性筛选及药效药理研究

实验目的：
通过体内小鼠原位瘤实验和基质胶埋植实验，原位肿瘤组织生长状况，检测新生血管形态学水平和病理学水平变化，评价ICJ抗肿瘤血管生成的体内药效活性。完成对体外ICJ抗肿瘤血管生成的体内验证与补充，为进一步机制研究提供依据。

课题概述（不超过200字）：
目前临床抗肿瘤转移的有效药物数目较少，抗转移药物研发成为肿瘤治疗的关键瓶颈问题。而瑞香狼毒自古被记载具有抗癌功效，是典型的的疗效明确、研发不足的抗癌中药。本课题着重从肿瘤免疫微环境入手，从肿瘤转移血管生成等角度，进一步分析瑞香狼毒有效成分-狼毒宁B的抗转移药效及分子机理，拓展瑞香狼毒的抗癌认识；促进对抗癌中药的资源开发和机理揭示，并为解决抗转移这一临床治疗难题提供可靠的实验室研究证据。

全部实验人员名单	序号	姓名	性别	上岗证号	职称	在本实验中承担的任务
	1	李琦	男	41907	副研究员	课题整体设计和实验方案的实施与指导
	2	杨庆	男	41910	副主任技师	原位瘤种植实验的实施
	3	翁小刚	男	41904	副研究员	动物实验的执行和辅助
	4	尹婕	女	2014071600079	在读硕士	实验动物的取材与检测
	5	陈颖	女	41905	副研究员	实验动物的取材
	6					

动物种属	品系	性别	体重（克）	数量	级别	饲养区域	实验时间	实验结束日期
大鼠属	Sprague–Dawley	雄	180-200	3	SPF	屏障环境	2015.7.1	2015.8.15
小鼠属	C57BL/6	雌	18-20	50只	SPF	屏障环境	2015.7.10	2015.7.24
小鼠属	BALB/c	雌	18-20	40只	SPF	屏障环境	2015.8.1	2015.9.1

实验方法	重点说明：1，动物实验过程，2 实验分组，3 申请动物数量是否恰当，4 实验过程中是否禁食禁水，其时间长度是多少，5 其它与动物福利伦理有关的问题。<br><br>SD大鼠饲养6周后，麻醉后，脱颈处死，取主动脉，去除结缔组织等切环，置于基质上培养。<br><br>在无菌环境下，按 1×10^4 4T1 细胞/只皮下注射入BALB/c乳腺脂肪垫，正常饮食，待接种部位可见瘤块，按平均瘤体积分为四组，每组10只，每日腹腔给药，26天后，麻醉后，脱颈处死，取原位瘤，进行免疫组化染色。<br><br>按随机数表法将C57/BL6分为五组，每组10只，在无菌环境下，将含或不含4T1细胞的0.5 ml基质胶/只皮下注射入C57/BL6腹部，正常饮食，每日腹腔给药，7天后，麻醉后，脱颈处死，取出基质胶，进行血管分析和免疫组化染色。
观察指标	原位瘤形态，免疫组化分析；<br>基质胶血红蛋白含量，免疫组化分析；<br>大鼠主动脉成环微血管MTT染色。
实验结束后处死动物的方法	麻醉后，颈椎脱臼处死法
实验过程中动物出现异常情况的处置方法	无
其它需要说明的内容	无

课题负责人意见：	同意	√	不同意		签　名
不同意的理由：					[illegible]<br>2015年6月24日
兽医师意见：	同意	√	不同意		签　名
不同意的理由：					[illegible]<br>2015年6月25日
实验动物福利伦理委员会主任意见：	同意	√	不同意		签　名
不同意的理由或建议修改的意见：					[illegible]<br>2015年6月26日

请在下列选项中打√（可以是单项，也可以是多项）

■ 开展的实验类型是：

☐ 开展___个月的长期毒性试验研究；

☐ 开展急性毒性试验研究；

☐ 开展药代动力学试验研究；

☑ 开展药效学实验研究；

☐ 开展其它科学研究：______________________________。

■ 处死动物的全过程是否是在麻醉条件下（动物不再苏醒）：☐ 是，☐ 不是；

☐ 实验过程中由于静脉采血，会对实验动物造成最低限度的动物紧张或压抑；

☐ 实验过程中由于静脉采血或静脉给予实验药物，需要对动物静脉埋入采血管或输入药物管，管道的埋入时间是：_____天；

☐ 实验过程中需要对动物施行外科手术或体内植入特定的实验物品或器具，该手术的时间是__2__小时，植入特定物品的时间是__7__天；

☐ 实验过程中需要对动物进行持续性束缚（不使用麻醉剂），以观察特定的实验指标，该持续性束缚的时间是：______小时；

■ 实验过程中需要对动物给予药物或实验物质（麻醉剂除外），其给药途径是：

☑ 口服、☐ 静脉、☐ 皮下、☐ 腹腔、☐ 皮肤、☐ 其它__________；

■ 给予的药物或物质是：

☐ 已知处方中药；

☐ 未知处方中药；

☑ 单一化学成分；

☐ 转基因物质__________；

☐ 经灭活的微生物加工产品__________；

☐ 中药处方中含有毒中药__________；

☐ 含放射性物质__________；

☐ 含重金属物质__________；

☐ 其它有害物质__________。

☐ 实验中采用的麻醉剂是：1%戊巴比妥钠__________；麻醉方式是：

☐ 全身麻醉；　☐ 吸入麻醉　☑ 注射麻醉

☐ 局部麻醉

☐ 本课题所用动物仅用于采血，实验结束后继续饲养，直至该动物用于其它实验（重新填写申请报告）；

■ 该项目是否必须使用动物进行实验：

☑ 必须使用动物；

☐ 可以不使用动物，如使用细胞，但科学意义会受到影响；

☐ 可以使用计算机模拟，但成本太高，且科学意义会受到影响。

☐ 其它需要说明的：

中国中医科学院中药研究所福利伦理委员会联系方式

秘　书：王丽芳

联系电话：(010)64284228，13693336098

电子邮箱：wanglifang73@163.com

地　址：北京市朝阳区樱花园东路甲 4 号中国中医科学院中药研究所动物房办公室
邮　编：100029
