# Supplementary figures and images for: Chamaejasmine B Induces the Anergy of Vascular Endothelial Cells to VEGFA Pro-angiogenic Signal by Autophagic Regulation of VEGFR2 in Breast Cancer
Source: Front Pharmacol. 2018 Jan 22;8:963. doi: 10.3389/fphar.2017.00963 (PMC5786572; doi:10.3389/fphar.2017.00963)

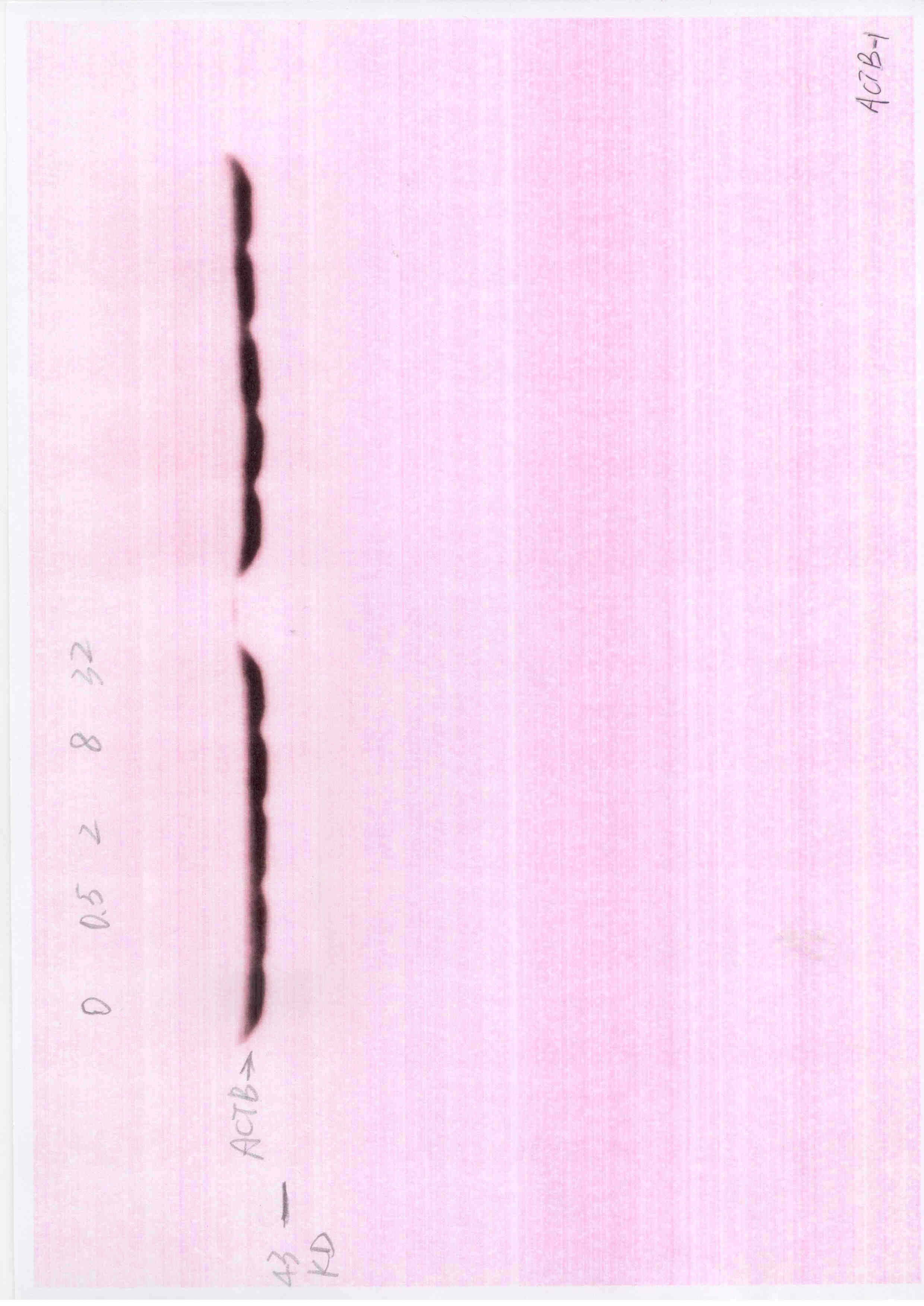

Supplement: Figure S1 — β-actin in Western blot of ICJ selectively induced autophagy of HUVEC. [file Image1.JPEG]

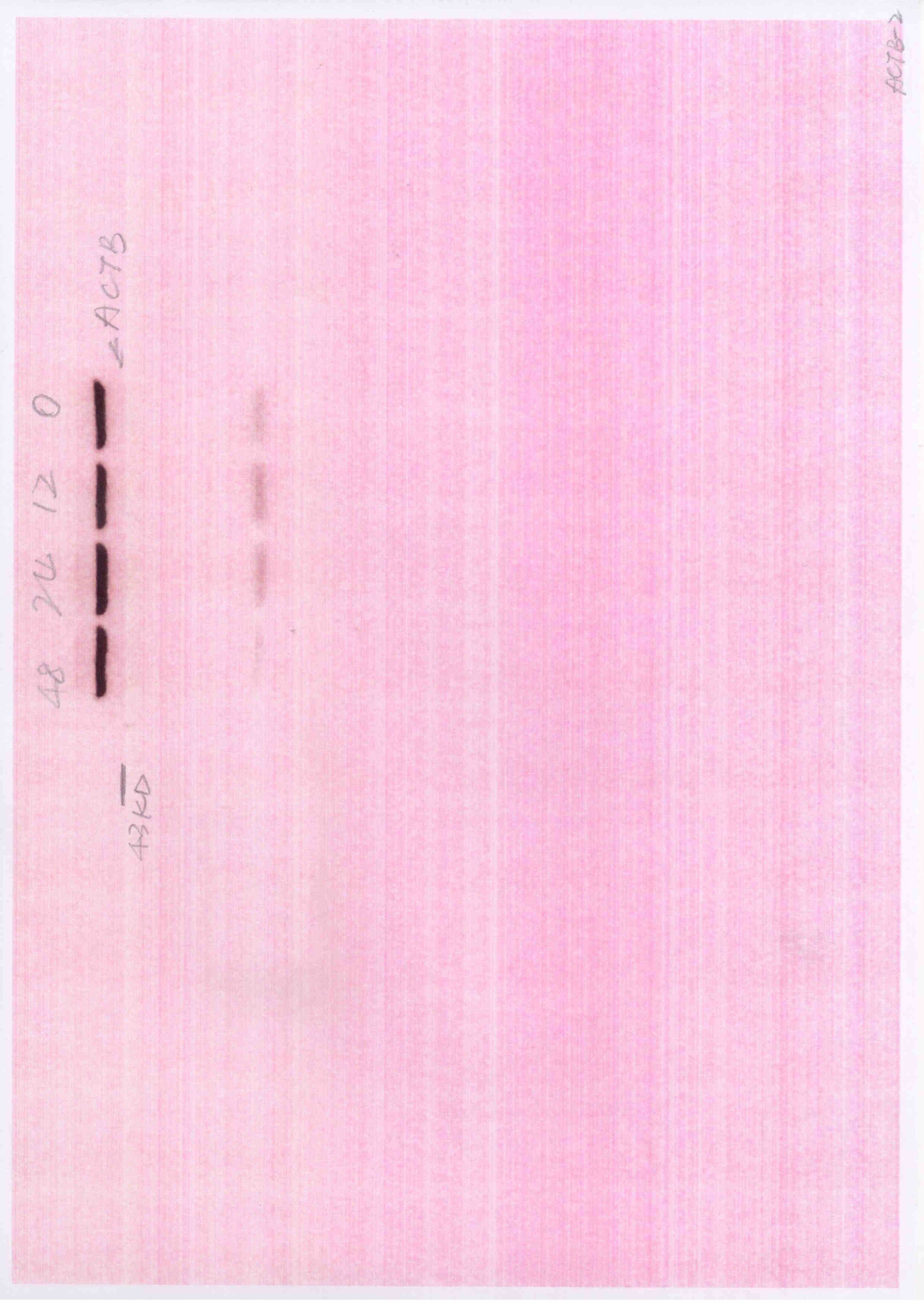

Supplement: Figure S2 — β-actin in Western blot of the blockage of autophagy rescues the ICJ-impaired angiogenesis. [file Image2.JPEG]

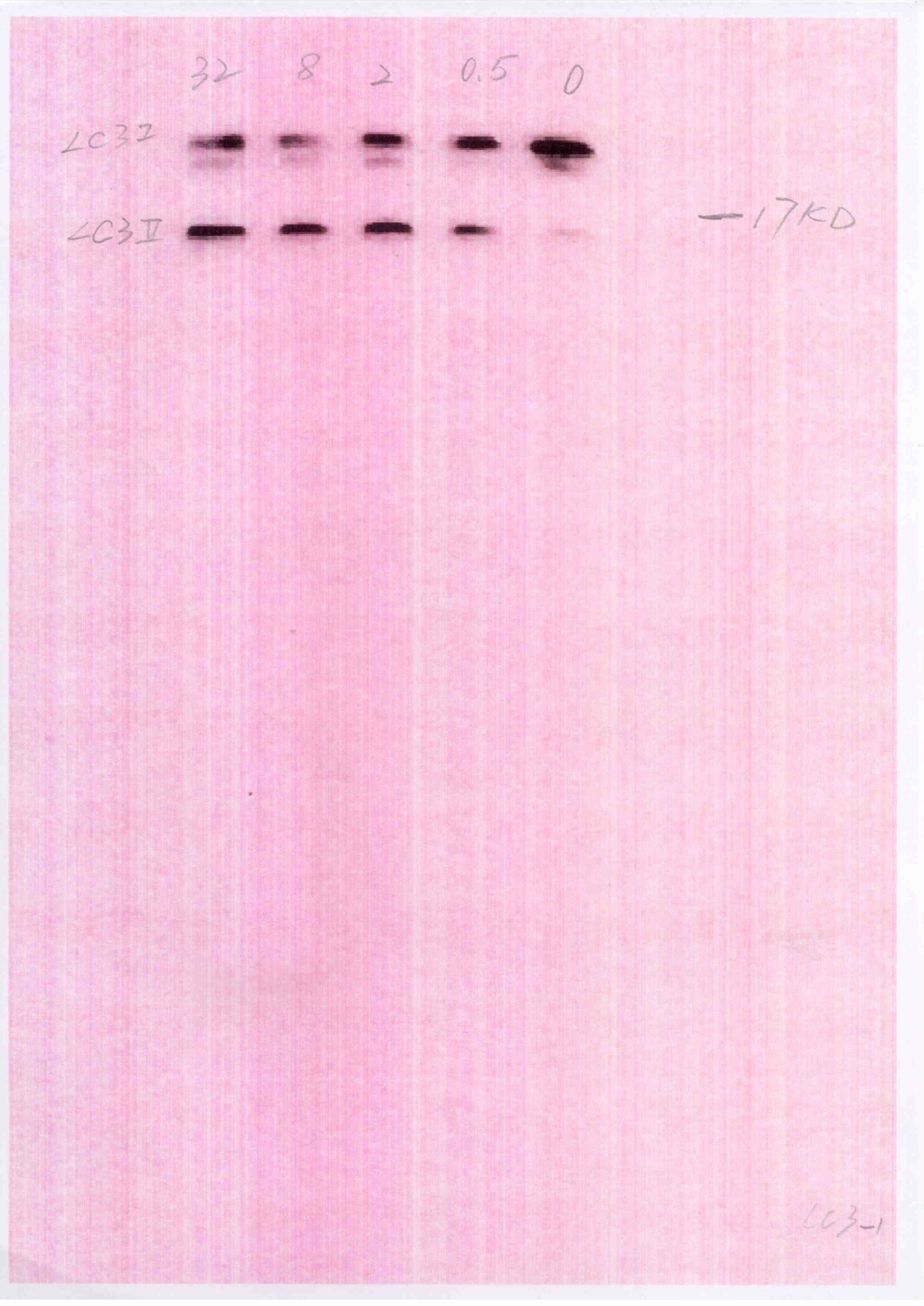

Supplement: Figure S3 — Detection of the autophagic biomarker, LC3I/II in HUVEC by Western blot analysis. [file Image3.JPEG]

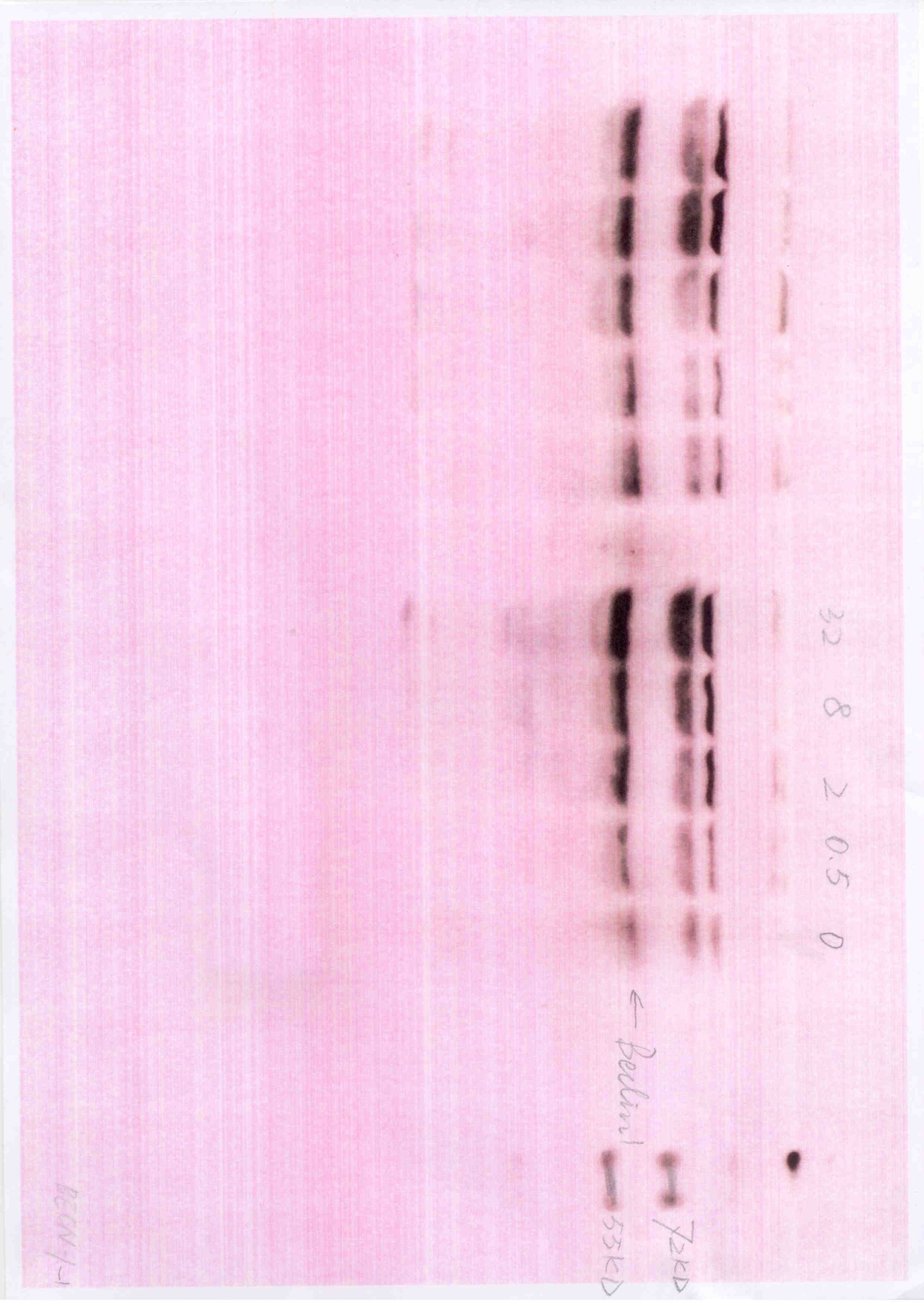

Supplement: Figure S4 — Beclin-1 in Western blot of ICJ selectively induced autophagy of HUVEC. [file Image4.JPEG]

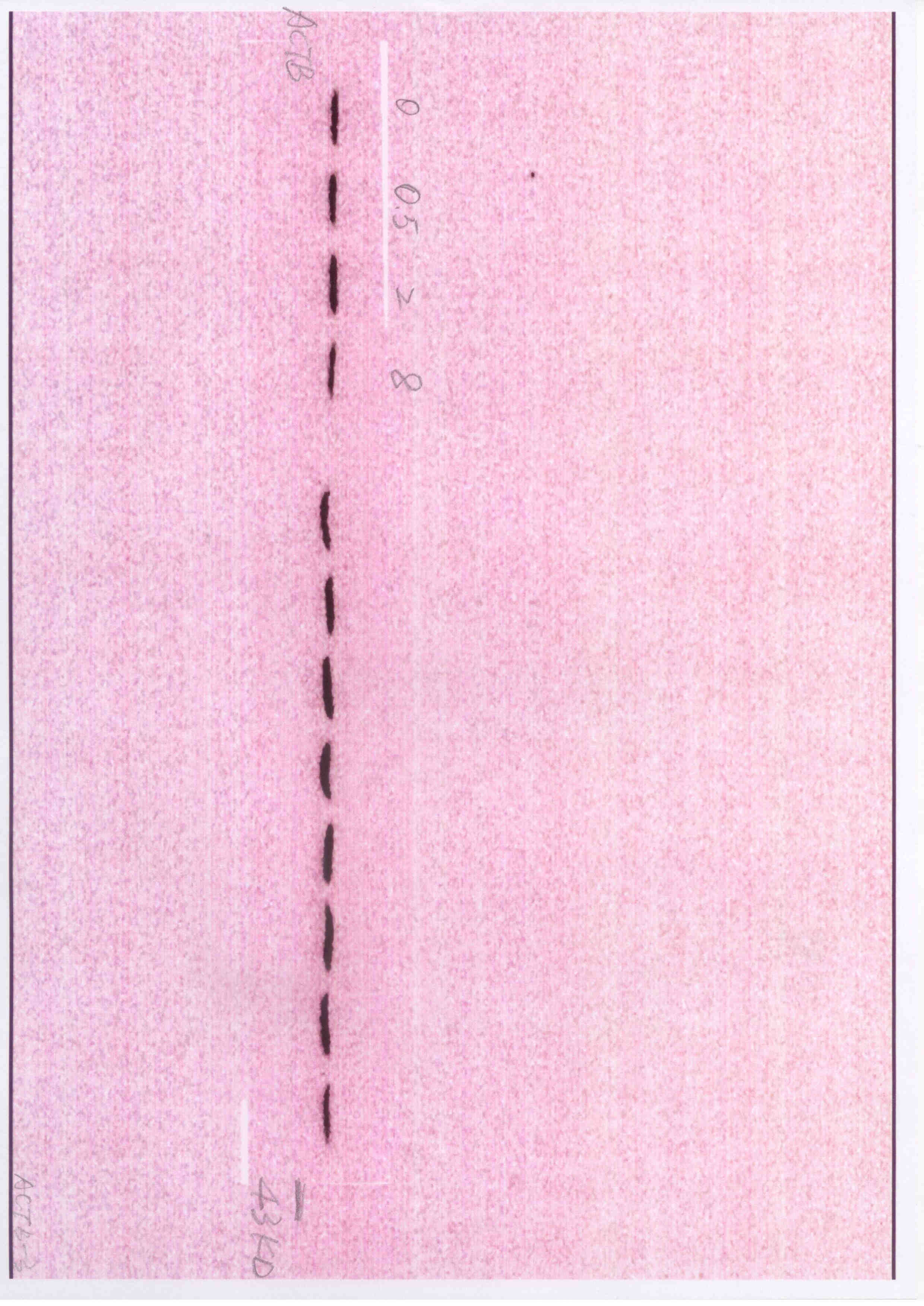

Supplement: Figure S5 — β-actin in Western blot analysis of VEGFR2 in tumor-HUVEC co-culture model pretreated of ICJ for 24 h. [file Image5.JPEG]

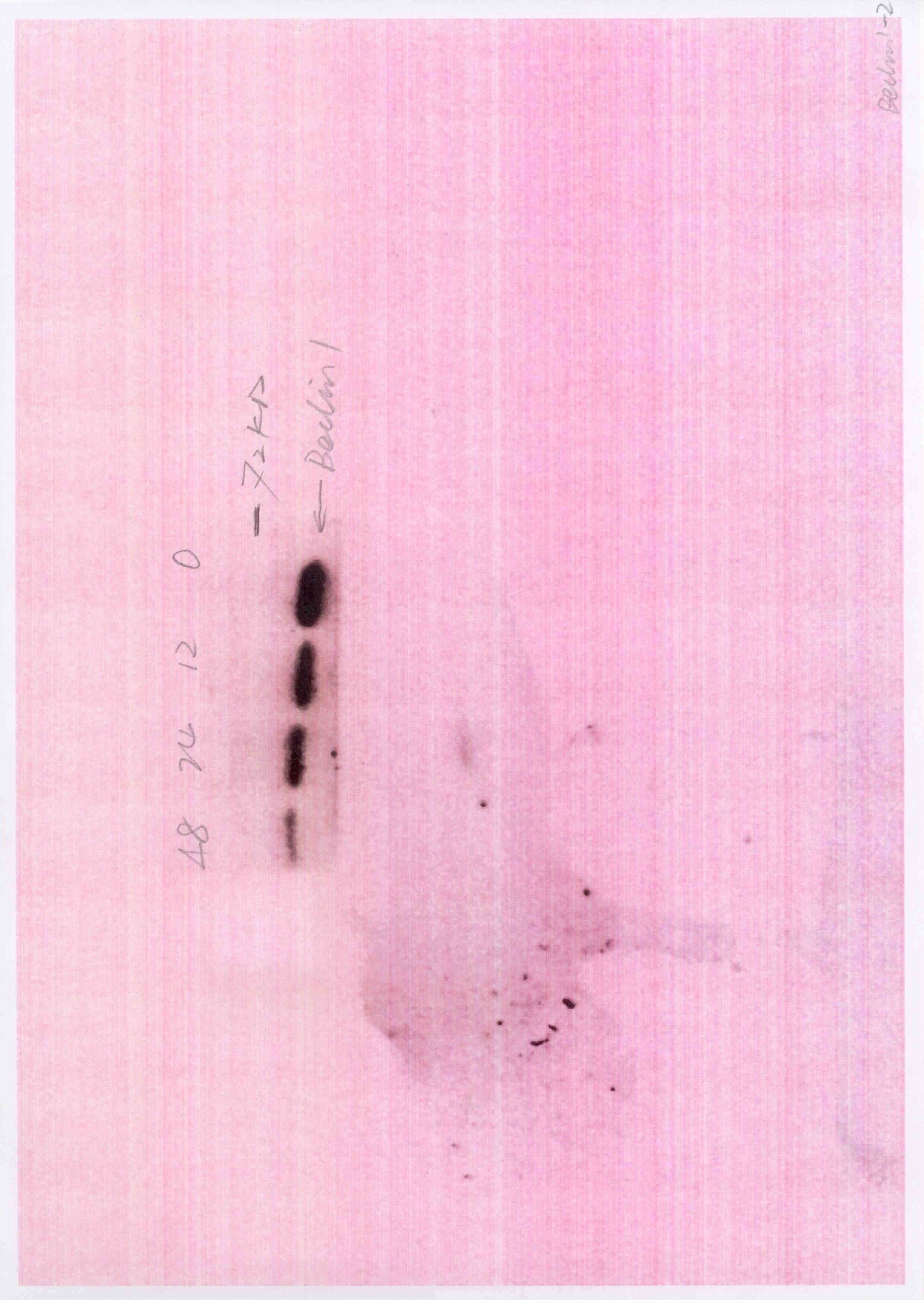

Supplement: Figure S6 — Silencing efficiency test of Beclin-1. Western blot showed that Beclin-1 expression was time-dependently silenced. [file Image6.JPEG]

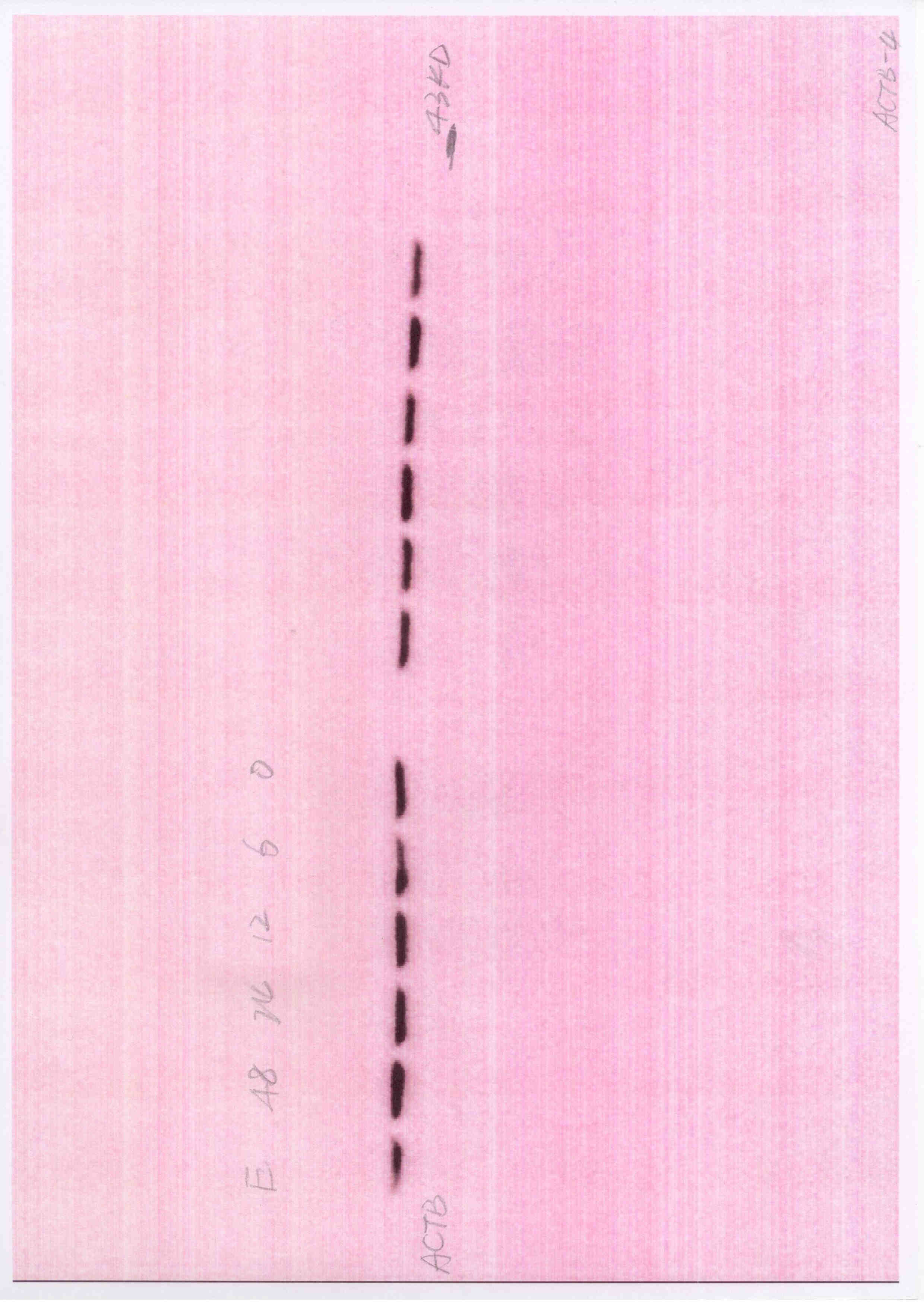

Supplement: Figure S7 — β-actin in Western blot analysis of time-course detection by 8 mg/ml of ICJ pretreatment. [file Image7.JPEG]

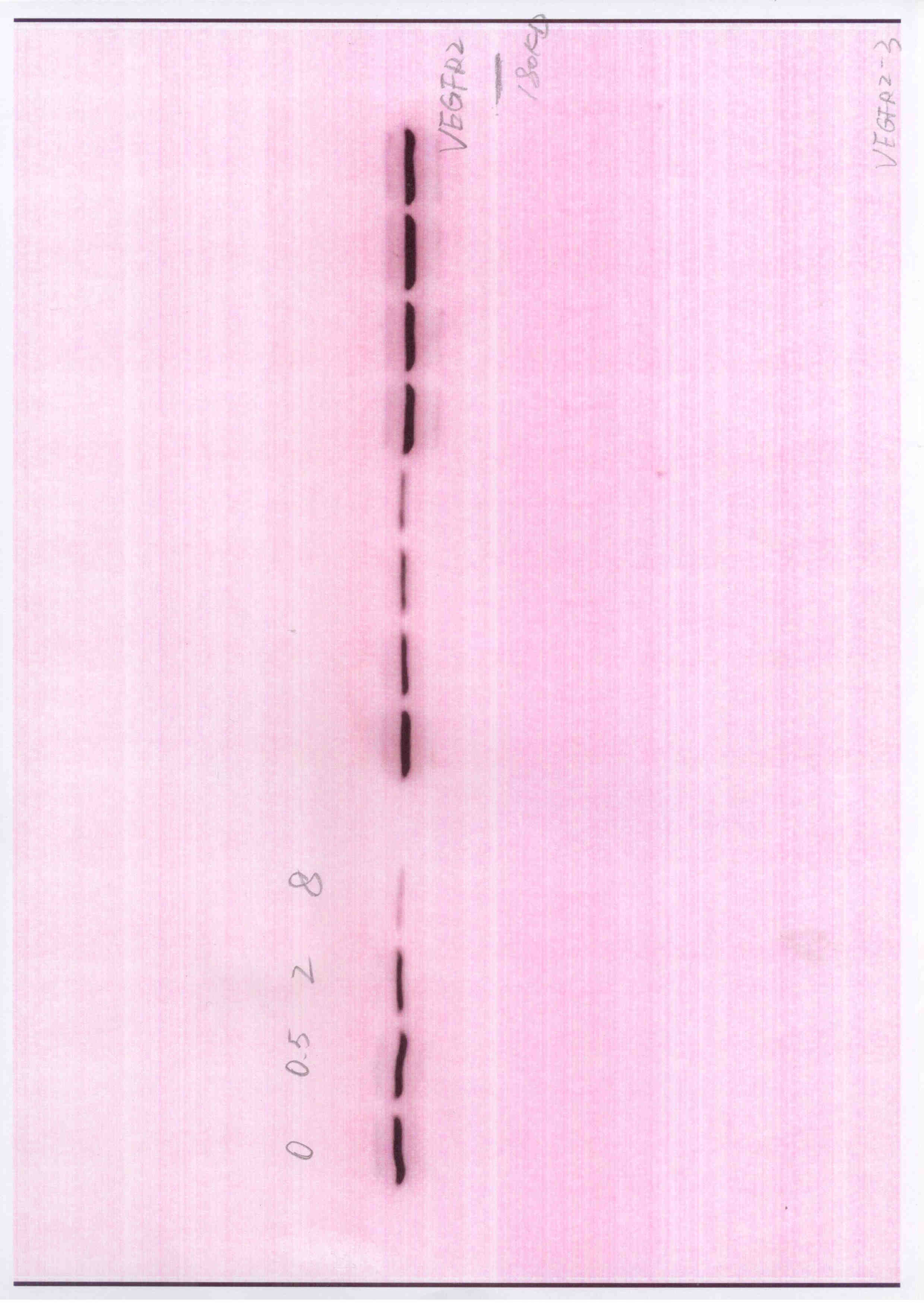

Supplement: Figure S8 — VEGFR2 in Western blot analysis of VEGFR2 in tumor-HUVEC co-culture model pretreated of ICJ for 24 h. [file Image8.JPEG]

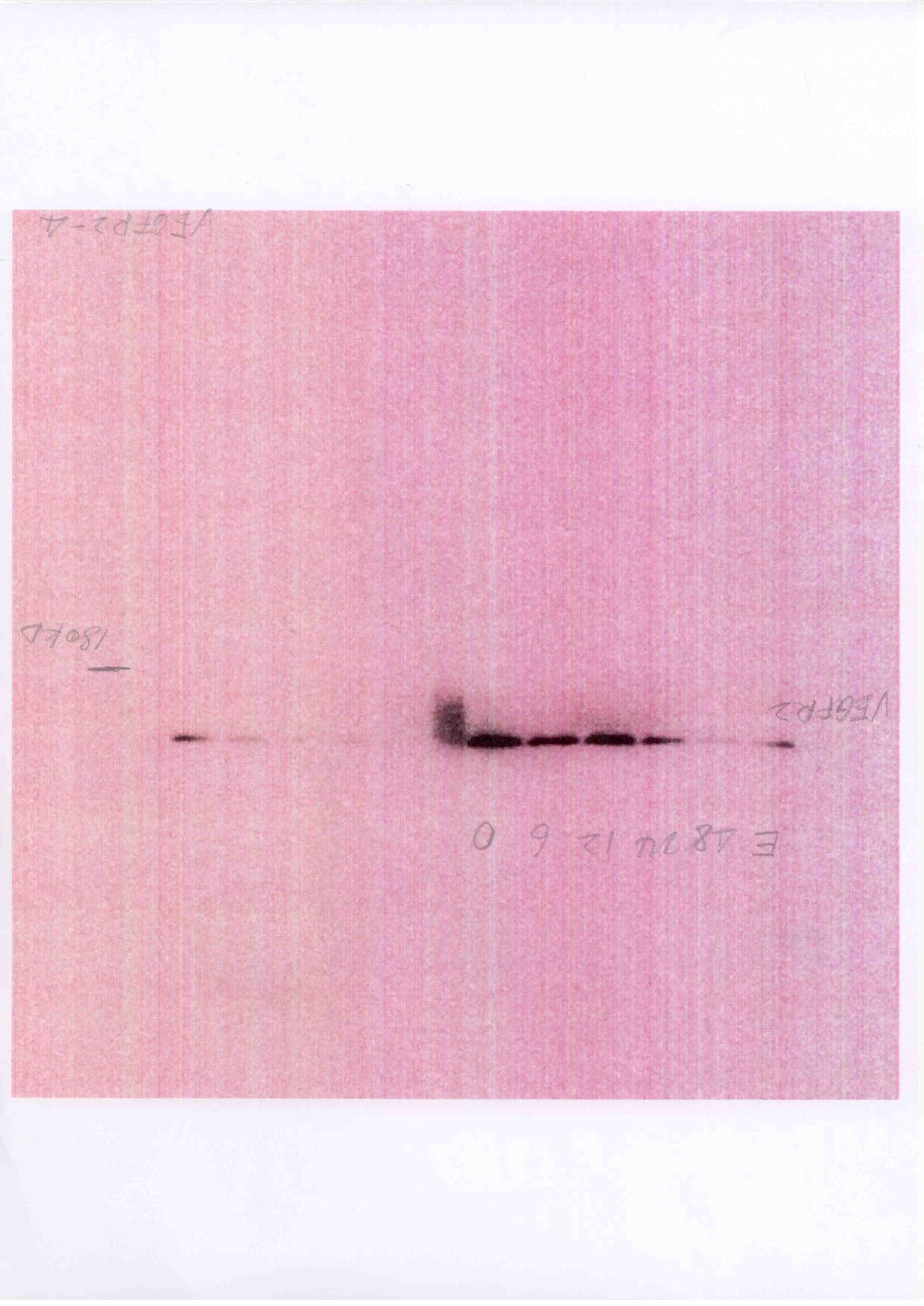

Supplement: Figure S9 — VEGFR2 in Western blot analysis of time-course detection of VEGFR2 by 8 mg/ml of ICJ pretreatment. [file Image9.JPEG]

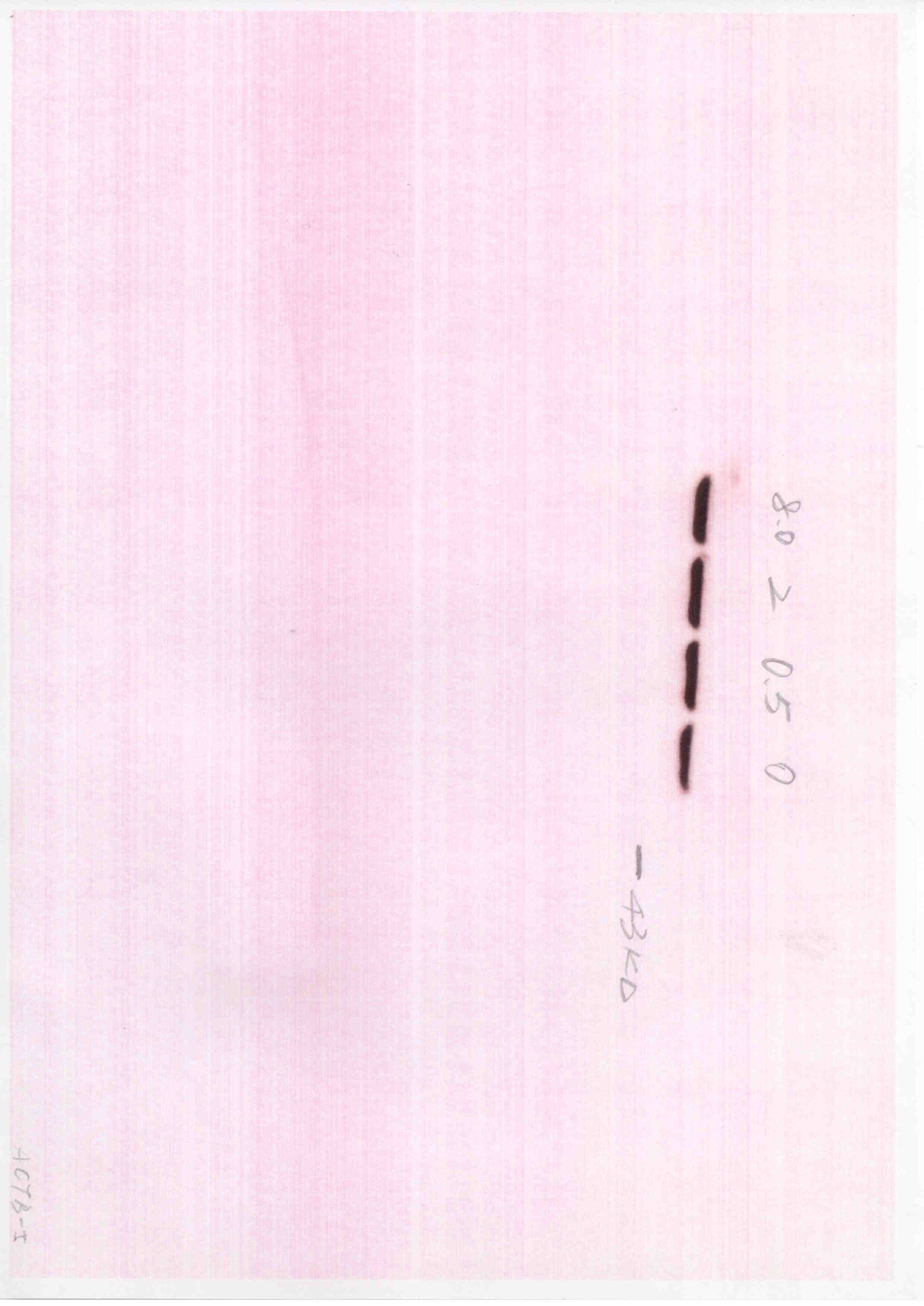

Supplement: Figure S10 — β-actin in Western blot analysis of co-immunoprecipitation analysis in HUVEC from co-culture model. [file Image10.JPEG]

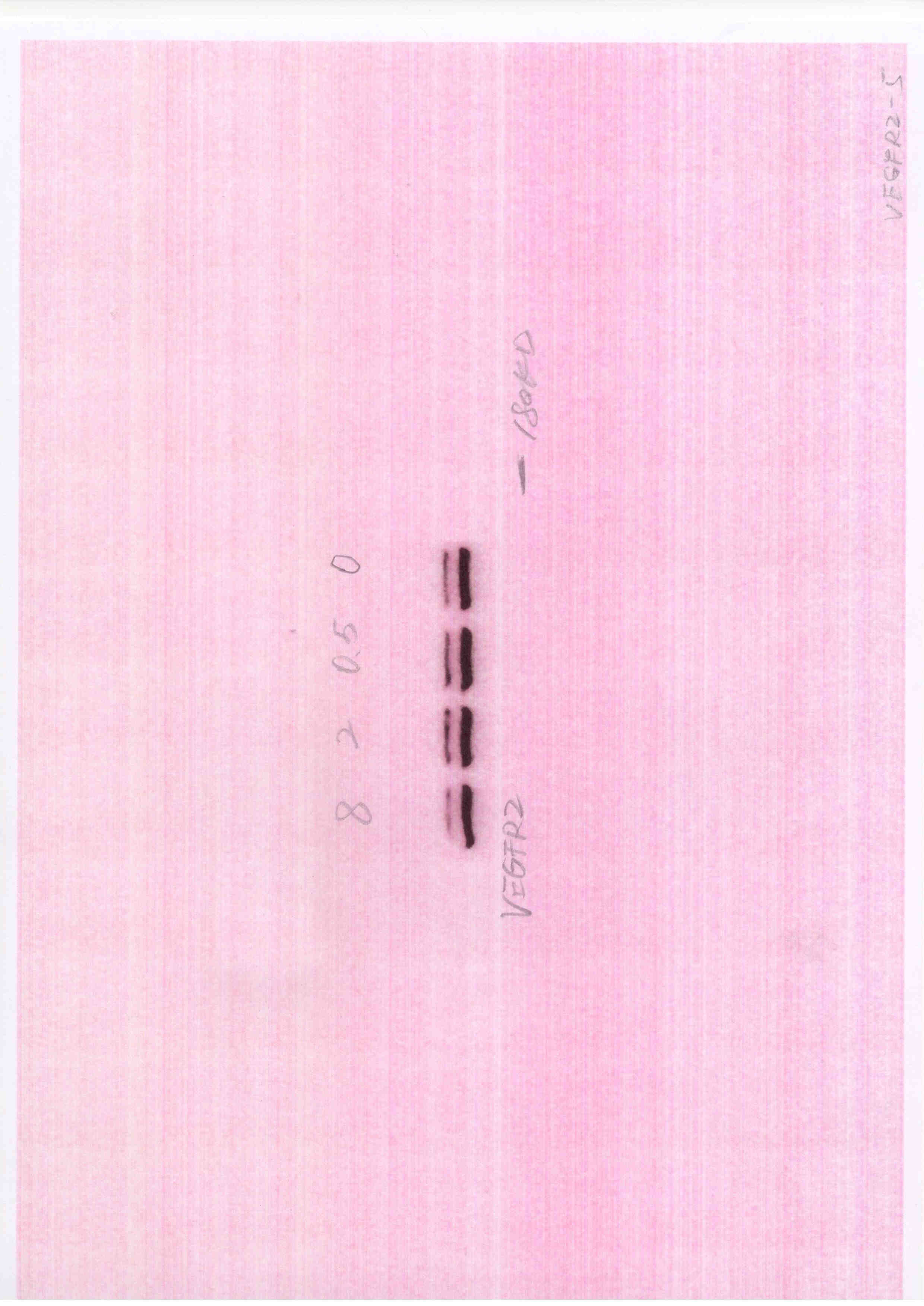

Supplement: Figure S11 — VEGFR2 in Western blot analysis of co-immunoprecipitation analysis in HUVEC from co-culture model. [file Image11.JPEG]

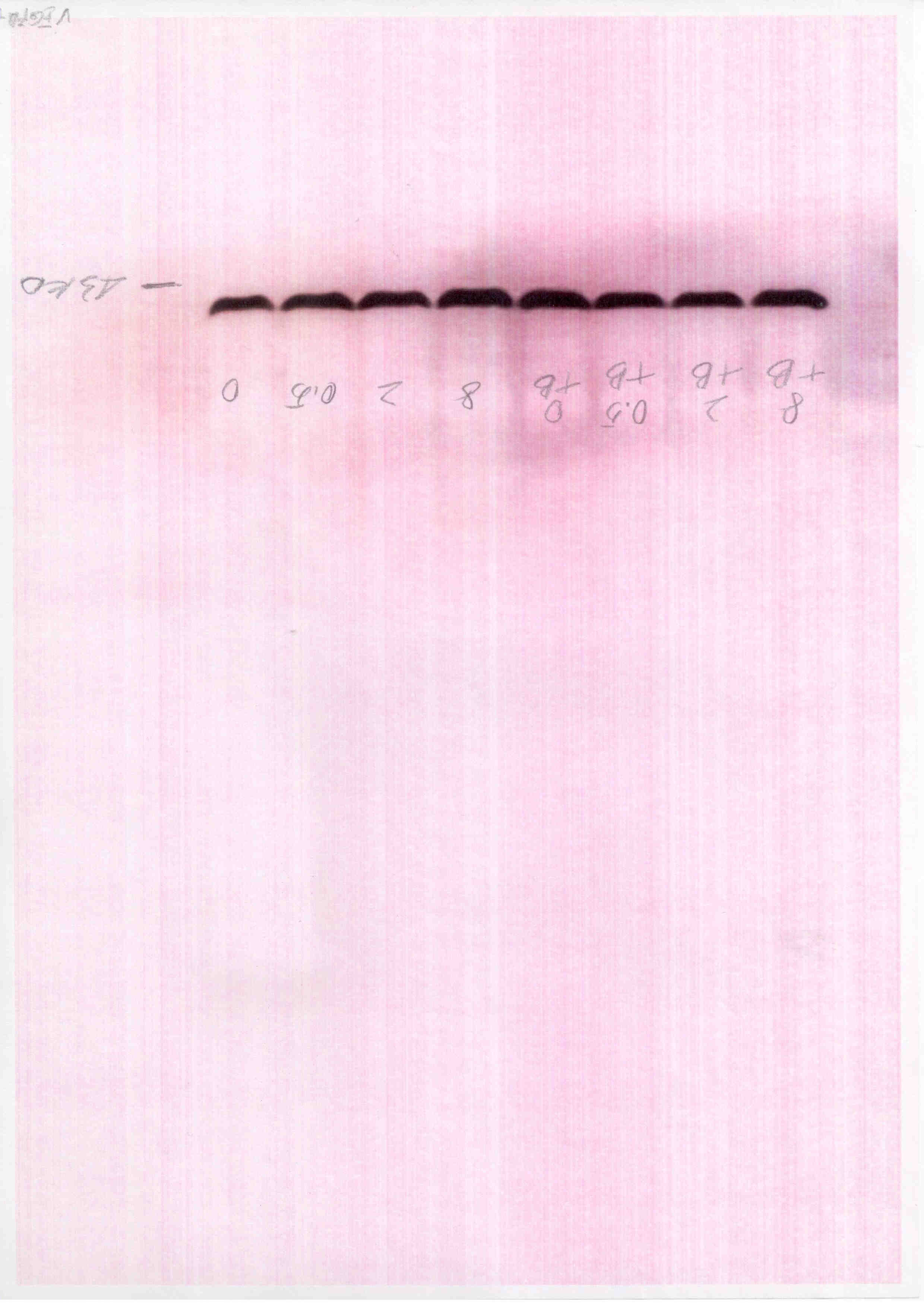

Supplement: Figure S12 — β-actin in Western blot analysis of BAF treated or non-treated HUVEC. [file Image12.JPEG]

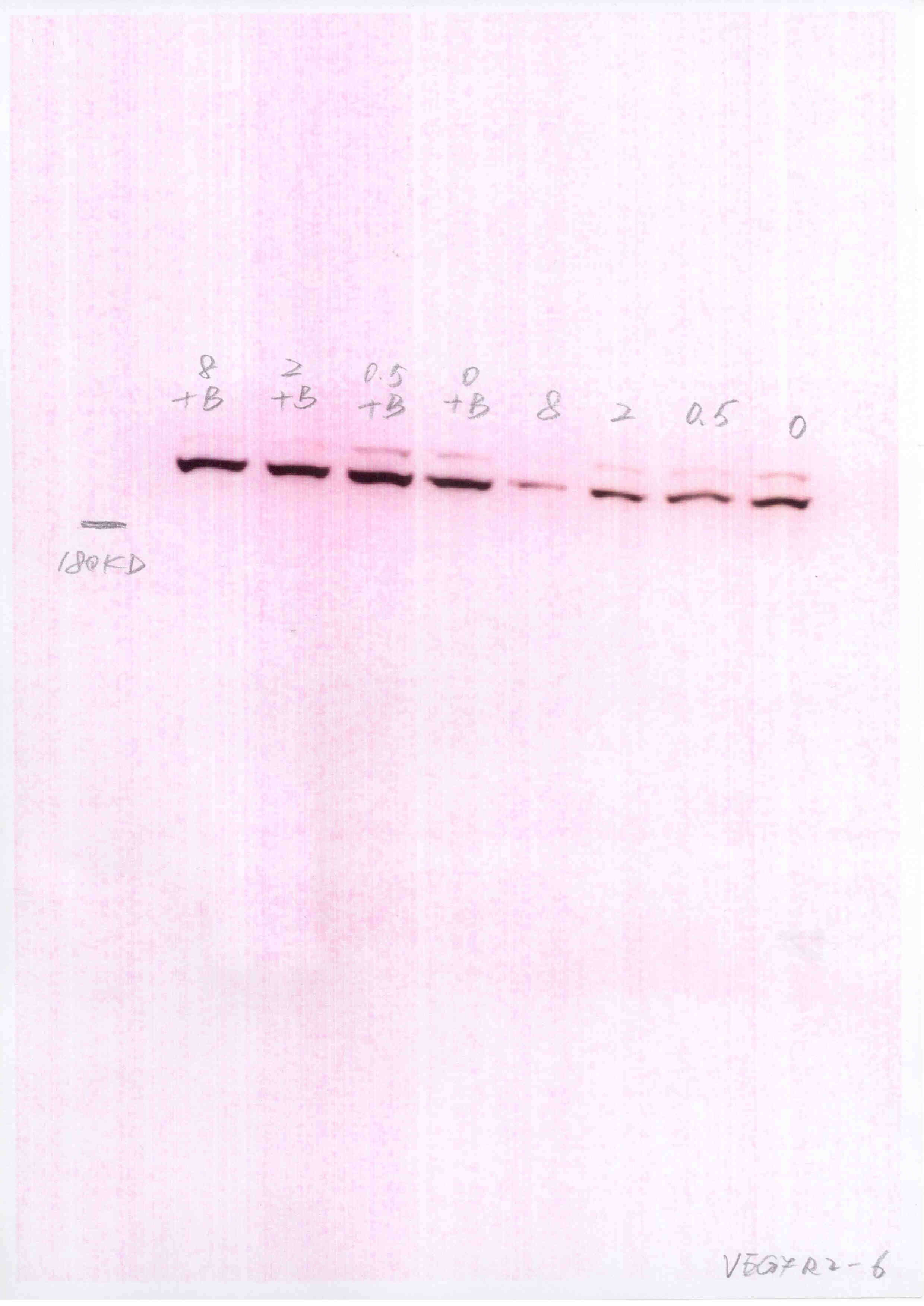

Supplement: Figure S13 — VEGFR2 in Western blot analysis of BAF treated or non-treated HUVEC. [file Image13.JPEG]
